# HSPA12A attenuates lipopolysaccharide-induced liver injury through inhibiting caspase-11-mediated hepatocyte pyroptosis via PGC-1α-dependent acyloxyacyl hydrolase expression

**DOI:** 10.1038/s41418-020-0536-x

**Published:** 2020-04-24

**Authors:** Jiali Liu, Shuya Du, Qiuyue Kong, Xiaojin Zhang, Surong Jiang, Xiaofei Cao, Yuehua Li, Chuanfu Li, Huaqun Chen, Zhengnian Ding, Li Liu

**Affiliations:** 1 Department of Geriatrics, Jiangsu Provincial Key Laboratory of Geriatrics, the First Affiliated Hospital of Nanjing Medical University, Nanjing, 210029 China; 2 Department of Anesthesiology, the First Affiliated Hospital of Nanjing Medical University, Nanjing, 210029 China; 3 Key Laboratory of Targeted Intervention of Cardiovascular Disease, Collaborative Innovation Center for Cardiovascular Disease Translational Medicine, Nanjing Medical University, Nanjing, 210029 China; 4 Departments of Surgery, East Tennessee State University, Johnson City, TN 37614 USA; 5 Jiangsu Key Laboratory for Molecular and Medical Biotechnology, College of Life Sciences, Nanjing Normal University, Nanjing, 210061 China

**Keywords:** Immune cell death, Infectious diseases

## Abstract

Liver dysfunction is strongly associated with poor survival of sepsis patients. Cytosolic lipopolysaccharide (LPS) sensing by Caspase-4/5/11 for pyroptosis activation is a major driver of the development of sepsis. Studies in macrophages and endothelial cells have demonstrated that LPS is inactivated by acyloxyacyl hydrolase (AOAH) and leading to desensitizing Caspase-4/5/11 to LPS. However, little is known about the cytosolic LPS-induced pyroptosis in hepatocytes during sepsis. Heat shock protein 12A (HSPA12A) is a novel member of the HSP70 family. Here, we report that LPS increased HSPA12A nuclear translocation in hepatocytes, while knockout of HSPA12A (*Hspa12a*^*−/−*^) in mice promoted LPS-induced acute liver injury. We also noticed that the LPS-induced Caspase-11 activation and its cleavage of gasdermin D (GSDMD) to produce the membrane pore-forming GSDMD^Nterm^ (markers of pyroptosis) were greater in livers of *Hspa12a*^*−/−*^ mice compared with its wild type controls. Loss- and gain-of-function studies showed that HSPA12A deficiency promoted, whereas HSPA12A overexpression inhibited, cytosolic LPS accumulation, Caspase-11 activation and GSDMD^Nterm^ generation in primary hepatocytes following LPS incubation. Notably, LPS-induced AOAH expression was suppressed by HSPA12A deficiency, whereas AOAH overexpression reversed the HSPA12A deficiency-induced promotion of LPS-evoked and Caspase-11-mediated pyroptosis of hepatocytes. In-depth molecular analysis showed that HSPA12A interacted directly with peroxisome proliferator-activated receptor γ coactivator 1α (PGC-1α) and increased its nuclear translocation, thereby inducing AOAH expression for cytosolic LPS inactivation, which ultimately leading to inhibition of the Caspase-11 mediated pyroptosis of hepatocytes. Taken together, these findings revealed HSPA12A as a novel player against LPS-induced liver injury by inhibiting cytosolic LPS-induced hepatocyte pyroptosis via PGC-1α-mediated AOAH expression. Therefore, targeting hepatocyte HSPA12A represents a viable strategy for the management of liver injury in sepsis patients.

## Introduction

Sepsis, a leading cause of death in intensive care units, is characterized by life-threatening organ dysfunction caused by a dysregulation of the host response to infection [1]. A number of organs are ultimately affected, but liver dysfunction develops early during sepsis, often on the day of diagnosis [2]. Because the liver plays important roles in the maintenance of metabolic and immune homeostasis, liver injury is strongly associated with poor outcome of sepsis patients. However, there is no specific therapy for this condition, which implies that a more comprehensive understanding of the pathogenesis of sepsis-induced liver injury is required.

A study from Intensive Care Over Nations showed that Gram-negative bacterial infections were more common than Gram-positive bacterial infections among patients with sepsis in the USA [3]. In response to infection, the multiple pathways are activated when highly conserved microbial pathogen-associated molecular patterns are recognized by pattern- recognition receptors. Lipopolysaccharide (LPS) is a major component of the outer membrane of Gram-negative bacteria and plays a critical role in sepsis by over-activating the innate immune system [4]. Extracellular LPS was thought to induce sepsis exclusively via the secretion of proinflammatory cytokines by binding to cell-surface toll-like receptor 4 (TLR4) [4]. However, the disappointing results of TLR4 inhibitors as anti-sepsis drugs in clinical trials indicate that a more important, TLR4-independent mechanism for LPS-induced injury may exist. Indeed, recent studies have shown that cytosolic LPS is a major driver of sepsis development and organ dysfunction without TLR4 requirement [4–6]. Therefore, promoting LPS turnover once endotoxin is delivered to the cytosol will diminish the LPS-induced septic injury. Cytosolic LPS is sensed by its intracellular receptors, Caspase-11 in rodents and Caspase-4/5 in humans, leading to their activation. Activated Caspase-4/5/11 cleaves gasdermin D (GSDMD) to generate an N-terminal fragment (GSDMD^Nterm^), which oligomerizes to form pores in the cytoplasmic membrane for the secretion of inflammatory mediators and ultimately causing regulated cell death, which is termed as pyroptosis [4, 5, 7, 8]. Thus, cleaved Caspase-11 and cleaved gasdermin D were considered as markers of pyroptosis. Therefore, desensitizing of cytosolic LPS by Caspase-4/5/11 has been proposed to be a potential therapeutic target for sepsis development [4].

The sensing of intracellular LPS by Caspase-4/5/11 has been shown to be determined by the six acyl chains in lipid A-moiety of intact LPS. Bexakis-acylated LPS fully activates, penta-acylated LPS weakly activates, and tetra-acylated LPS does not activate Caspase-11 [4, 9]. Intriguingly, the secondary (acyloxyacyl-linked) fatty acids of LPS can be removed by acyloxyacyl hydrolase (AOAH), a host enzyme, and leading to LPS biologically inactivated and unable to be sensed by Caspase-4/5/11 [10, 11]. Indeed, studies conducted in endothelial cells and macrophages have shown that AOAH deficiency exacerbated LPS-induced lung injury, whereas appropriate AOAH activity is associated with effective host defense against bacterial LPS [12, 13]. However, little is known regarding the role of AOAH in sepsis-induced hepatocyte injury or the regulation of AOAH expression.

Heat shock protein A12A (HSPA12A) is an atypical member of the heat shock protein 70 family [14]. We have recently demonstrated that HSPA12A is required for cerebral protection and obesity development [15, 16], and in particular, that HSPA12A is involved in the development of high-fat diet-induced non-alcoholic fatty liver disease [17], suggesting that it plays a role in the regulation of hepatic homeostasis. However, the role of HSPA12A in sepsis-induced organ injury, including liver dysfunction, has not been established.

Here, we report that HSPA12A protein translocated to the nucleus of hepatocytes following LPS treatment, while knockout of HSPA12A (*Hspa12a*^*−/−*^) in mice exacerbated LPS-induced acute liver injury. Further molecular analysis showed that the hepatoprotection of HSPA12A was mediated by inhibiting cytosolic LPS-induced pyroptosis of hepatocytes via peroxisome proliferator-activated receptor γ coactivator 1α (PGC-1α)-mediated AOAH expression.

## Results

### HSPA12A demonstrates nuclear translocation in hepatocytes following exposure to LPS

**Fig. 1:**
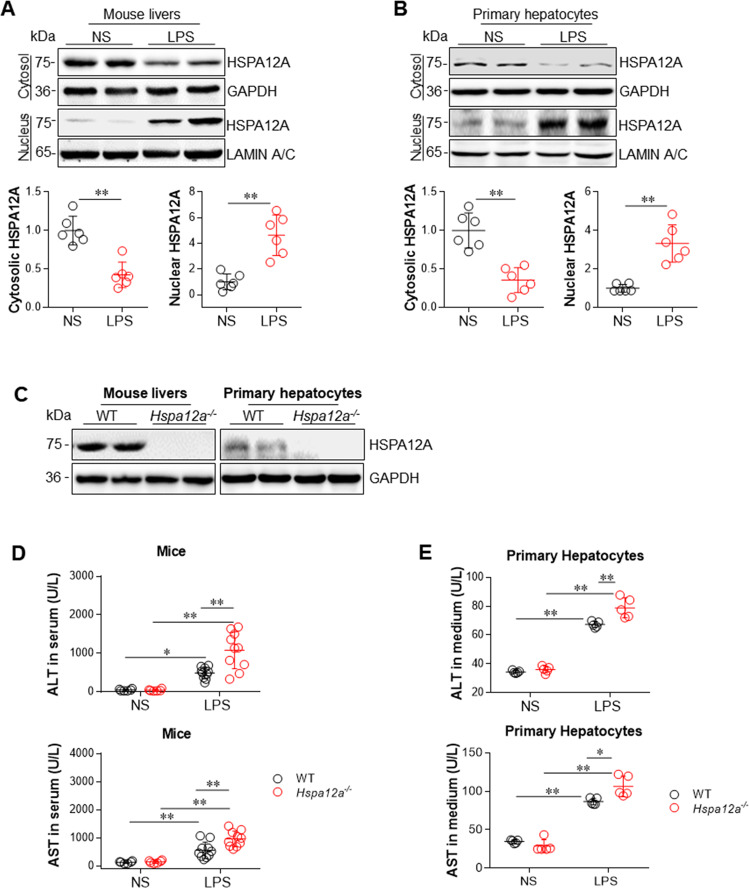
HSPA12A deficiency promoted the LPS-induced hepatic injury. **a** HSPA12A protein expression in livers. Cytosolic and nuclear protein fractions were prepared from mice livers following treatment with LPS or normal saline (NS) for 6 h. HSPA12A expression was analyzed by immunoblotting analysis. Blots for GAPDH or Lamin A/C served as loading controls. Data are mean ± SD, **P < 0.01 by Student’s two-tailed unpaired *t* test. *n* = 6/group. **b** HSPA12A protein expression in primary hepatocytes. Cytosolic and nuclear protein fractions were prepared from primary hepatocytes following incubation with LPS or NS for 6 h. HSPA12A expression was analyzed by immunoblotting analysis. Blots for GAPDH or Lamin A/C served as loading controls. Data are mean ± SD, **P < 0.01 by Student’s two-tailed unpaired *t* test. *n* = 6/group. **c** HSPA12A expression in *Hspa12a*^*−/−*^ mice. HSPA12A expression was examined in mice livers and isolated primary hepatocytes using immunoblotting. Note that HSPA12A expression was absent in livers and hepatocytes of *Hspa12a*^−/−^ mice. *n* = 10 mice/group. WT, wild type; *Hspa12a*^*−/−*^, HSPA12A knockout. **d** ALT and AST activities in mouse serum. Mice were treated with LPS or NS for 6 h. Serum was collected for ALT and AST activity measurements. Data are mean ± SD, *P < 0.05 and **P < 0.01 by two-way ANOVA followed by Tukey’s test. *n* = 6 for each NS group and *n* = 10 for each LPS group. **e** ALT and AST activities in culture medium. Primary hepatocytes were incubated with LPS or NS for 6 h. Culture medium was collected for ALT and AST activity measurements. Data are mean ± SD, *P < 0.05 and **P < 0.01 by two-way ANOVA followed by Tukey’s test. *n* = 5/group.

To evaluate the possible involvement of HSPA12A in sepsis-induced liver injury, we measured HSPA12A expression in the livers of mice following LPS challenge. The dose-effect of LPS on hepatic HSPA12A protein abundance was examined by challenge with LPS at dosages of 2.5, 5, 10, and 20 mg/kg for 6 h. HSPA12A protein levels were significantly lower in the cytosolic but higher in the nuclear fractions of mouse livers following challenge with LPS at dosages of 5, 10, and 20 mg/kg, respectively, than those of the normal saline (NS)-treated control mice (Figs. 1a, S1a). We therefore selected 5 mg/kg of LPS to challenge mice for different durations (3, 6, 12, and 24 h), and found that HSPA12A protein levels were rapidly decreased in the cytosolic but increased in the nuclear fractions after challenge with LPS for 6 h, respectively, when compared with those of the NS controls (Figs. 1a, S1b).

To determine whether these changes of HSPA12A occurred in liver hepatocytes, HSPA12A expression was measured in primary hepatocyte cultures following incubation with LPS for 6 h at different dosages (250, 500, 1000, and 2000 ng/ml). Similarly to the in vivo findings, incubation with LPS reduced the abundance of HSPA12A in the cytosolic but increased it in the nuclear fractions of the primary hepatocytes and peaked at 500 ng/ml of dosage (Figs. 1b, S1c). We thus selected 500 ng/ml LPS to challenge primary hepatocytes for different durations (3, 6, 12, and 24 h), and found that HSPA12A protein levels were lower in the cytosolic but higher in the nuclear fractions after LPS challenge and peaked at 6 h, respectively, when compared with those of the NS controls (Figs. 1b, S1d). Hepatocytes *Hspa12a* mRNA expression was not affected by LPS treatment (Fig. S2). These data show that hepatocyte HSPA12A undergoes nuclear translocation following LPS exposure.

Based on these findings, we selected 5 mg/kg LPS to treat mice and 500 ng/ml LPS to treat primary hepatocytes for 6 h in the following experiments. Mice treated with LPS (5 mg/kg) for 6 h exhibited reduction of body temperature and animal activity, decrease of systolic blood pressure, decreases of arterial blood oxygen saturation (SO_2_) and partial pressure of blood oxygen (pO_2_) whereas increased partial pressure of blood carbon dioxide pressure (pCO_2_), and increase of urea-nitrogen (Urea) (Fig. S3a–e) These changes suggest that mice treated with LPS (5 mg/kg) for 6 h undergoes a septic shock response. No mice died during experiments (Fig. S3f). Moreover, the liver injury, which indicated by increases of alanine transaminase (ALT) and aspartate transaminase (AST) activities in serum and activation of Caspase-11 in livers, were also significantly increased following treatment with LPS at 5 mg/kg of dosage for 6 h (Fig. S4a–d). Also, ALT and AST activities in culture medium and Caspase-11 activation in hepatocytes were significantly increased in primary hepatocyte cultures following incubation with 500 ng/ml LPS for 6 h (Fig. S5a–d).

### HSPA12A deficiency promotes LPS-induced injury in both mouse liver and primary hepatocytes

We next determined whether HSPA12A is required for the development of LPS-induced liver injury using *Hspa12a*^*−/−*^ mice, in which HSPA12A expression is absent in the liver and the derived primary hepatocytes (Fig. 1c). To evoke LPS-induced sepsis, mice were administrated with LPS for 6 h. LPS increased ALT and AST activities in the serum of mice of both genotypes *versus* their respective NS-treated controls (Fig. 1d). However, the LPS-induced increases in serum ALT and AST activities were greater in *Hspa12a*^*−/−*^ mice than in WT controls. When LPS challenge up to 24 h, *Hspa12a*^*−/−*^ mice still demonstrated higher serum ALT and AST activities than WT mice (Fig. S6). We also found that *Hspa12a*^*−/−*^ mice demonstrated lower body temperature and activities than those in WT mice following LPS treatment (Fig. S7).

To determine whether the exacerbated liver injury in *Hspa12a*^*−/−*^ mice is directly attributable to hepatocyte damage, we isolated primary hepatocytes from *Hspa12a*^*−/−*^ and WT mice (Fig. 1c, right panels). Following LPS exposure for 6 h, increases in ALT and AST activities were detected in cell culture medium of both genotypes (Fig. 1e). However, the LPS-induced increases in ALT and AST activities were larger in the medium of *Hspa12a*^*−/−*^ hepatocytes than in WT controls, suggesting that HSPA12A deficiency exacerbated the LPS-induced ALT and AST leakage from hepatocytes. Taken together, these data indicate that HSPA12A is required for hepatoprotection against LPS challenge.

To investigate whether the exacerbated hepatic injury in *Hspa12a*^*−/−*^ mice is a primary event, we examined injuries in other organs according to previous studies [18, 19]. Arterial blood gas analysis showed that LPS significantly decreased pO_2_ and SO_2_ in both genotypes (Fig. S8a). The pCO_2_ was remained unchanged in WT mice but increased in *Hspa12a*^*−/−*^ mice following LPS challenge. Though *Hspa12a*^*−*^^*/−*^ mice showed trends of decrease in pO_2_ and SO_2_ and increase in pCO_2_ compared with WT mice, the differences did not reached a statistical significance. LPS increased blood Urea levels in both genotypes; however, no difference of Urea levels was detected between WT and *Hspa12a*^*−*^^*/−*^ mice following LPS treatment (Fig. S8b). Histological examination of spleen showed that LPS caused hyperemia, increased leukocyte infiltration in the red pulp, and increased percentage of white pulp in both genotypes; however, the LPS-induced histological abnormalities were comparable between WT and *Hspa12a*^*−*^^*/−*^ mice (Fig. S8c). LPS also caused histological abnormalities in intestines, like an increase in leukocyte infiltration in lamina propria and a decrease in finger-like villus projection in both genotypes; however, the LPS-induced abnormalities were comparable between genotypes (Fig. S8d).

### HSPA12A deficiency increases LPS accumulation in hepatocytes

**Fig. 2:**
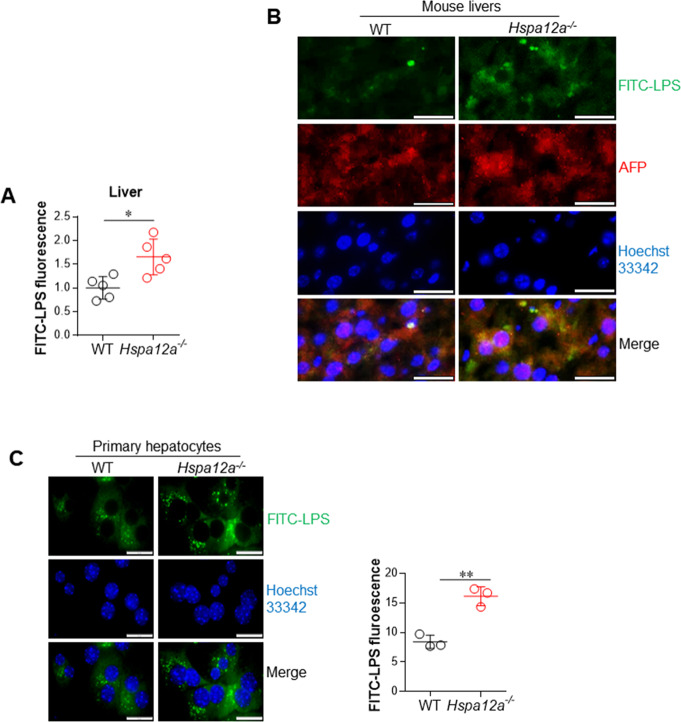
HSPA12A deficiency increased intra-hepatocyte LPS abundance following LPS exposure. **a** LPS contents in liver homogenates. 6 h after FITC-LPS treatment, liver tissues were collected from mice. LPS abundance in liver homogenates (unites/mg tissue, relative) was indicated by the FITC fluorescence intensity that measured by a fluorometer at excitation/emission wavelengths of 490/530 nm. Data are mean ± SD, *P < 0.05 by Student’s two-tailed unpaired *t* test. *n* = 5/group. **b** LPS in cytoplasm of hepatocytes of mouse livers. Frozen liver sections were prepared 6 h after FITC-LPS treatment. The sections were immunostained with alpha-fetoprotein (AFP), and Hoechst33342 was used to counter stain nuclei. Note that more FITC-LPS was present in hepatocyte cytoplasm of *Hspa12a*^*−/−*^ mouse livers. Scale bar = 20 μm. *n* = 4/group. **c** LPS in cytoplasm of primary hepatocytes. After incubation with FITC-LPS for 6 h, the primary hepatocytes were counter stained with Hoechst33342. The staining was observed and quantified using a fluorescence microscope. Data were expressed as FITC fluorescence intensity *10^3^/mm^2^ cell area. Data are mean ± SD, **P < 0.01 by Student’s two-tailed unpaired *t* test. *n* = 3/group. Scale bar = 20 μm.

Cytosolic LPS has been proposed to be a major initiator of cell damage during sepsis/septic shock [4, 5, 20]. Because the liver is the principal organ responsible for clearing circulating LPS and hypotacyte import a substantial quantity of LPS [21–23], we determined the effect of HSPA12A deficiency on LPS deposition in the liver in vivo and primary hepatocytes in vitro. Following administration of FITC-conjugated LPS (FITC-LPS) for 6 h, *Hspa12a*^*−/−*^ mice had higher serum and liver LPS contents than WT mice (Figs. 2a, S9). Furthermore, immunostaining demonstrated the presence of FITC-LPS in AFP-positive cells in livers of mice of both genotypes, but to a greater contents in *Hspa12a*^*−/−*^ livers, suggesting that HSPA12A deficiency exacerbated the accumulation of LPS in mouse hepatocytes (Fig. 2b). Consistent with this finding, the in vitro experiments demonstrated greater intracellular accumulation of LPS in *Hspa12a*^*−/−*^ primary hepatocytes than in WT control following incubation with FITC-LPS for 6 h (Fig. 2c), and the accumulation showed a time-dependent manner (Fig. S10). To determine whether intracellular LPS was free in cytosol or within intracellular vesicles, we performed immunostaining for an intracellular vesicle marker Flotillin-1 according to previous studies [24]. We found that predominant FITC-LPS was not within intracellular vesicles (Fig. S11). Collectively, these data suggest that HSPA12A deficiency increases intra-hepatocyte LPS accumulation following LPS exposure.

### HSPA12A deficiency promotes hepatocyte pyroptosis following LPS challenge

**Fig. 3:**
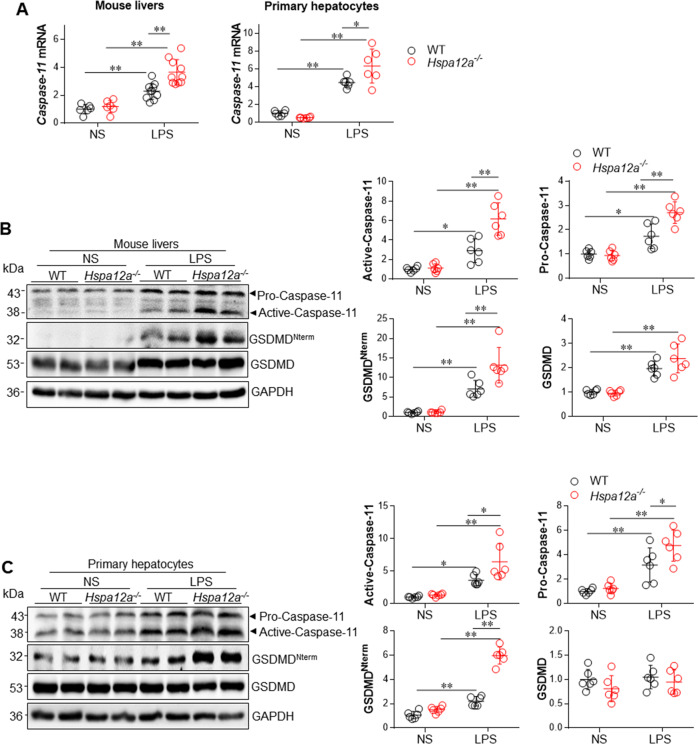
HSPA12A deficiency promoted Casnpase-11-mediated pyroptosis in both mouse livers in vivo and primary hepatocytes in vitro. **a** Liver tissues were collected from mice 6 h after LPS or normal saline (NS) treatment. In another set of experiments, cultured primary hepatocytes were collected after LPS or NS incubation for 6 h. The following analysises were performed. *Caspase-11* mRNA expression was evaluated using real-time PCR. Data are mean ± SD, *P < 0.05 and **P < 0.01 by two-way ANOVA followed by Tukey’s test. *n* = 6–10/group. Caspase-11 activation and GSDMD cleavage was examined in livers (**b**) and primary hepatocytes (**c**) using immunoblotting. Blots for GAPDH served as loading controls. Data are mean ± SD, *P < 0.05 and **P < 0.01 by two-way ANOVA followed by Tukey’s test. *n* = 6/group.

Because cytosolic LPS can be sensed by its intracellular receptor Caspase-11, which induces pyroptosis in mice [5, 8, 25], we next determined the effects of HSPA12A deficiency on Caspase-11-mediated pyroptosis in the liver. We found that LPS upregulated *Caspase-11* mRNA expression in the livers of mice of both genotypes, but this LPS-induced upregulation was more marked in livers of *Hspa12a*^*−/−*^ mice than in those from WT controls (Fig. 3a, left panel). At the protein levels, mouse livers of both genotypes showed greater Caspase-11 activation and its cleavage of GSDMD to produce the active membrane pore-forming GSDMD^Nterm^ peptide (markers of pyroptosis) following LPS treatment (Fig. 3b). However, the LPS-induced Caspase-11 activation and GSDMD^Nterm^ peptide generation were more marked in *Hspa12a*^*−/−*^ livers than in WT livers (Fig. 3b).

LPS has been shown to induce pyroptosis in endothelial cells, macrophages, and splenic cells [5, 8, 25]; however, little is known about this process in hepatocytes. To determine whether hepatocytes respond to cytoplasmic LPS for initiating Caspase-11-mediated pyroptosis and whether HSPA12A influences this process, we performed experiments in primary hepatocytes. We found that the LPS-induced upregulation of *Caspase-11* mRNA was greater in *Hspa12a*^*−/−*^ than WT hepatocytes (Fig. 3a, right panel). Similarly, the LPS-induced Caspase-11 activation and GSDMD^Nterm^ peptide production (markers of pyroptosis) was more marked in HSPA12A-deficient hepatocytes (Fig. 3c). The exacerbation of LPS-induced pyroptosis in *Hspa12a*^*−/−*^ hepatocytes was confirmed by the increase of lactic acid dehydrogenase (LDH) leakage compared withWT controls (Fig. S12). Taken together, these data suggest that HSPA12A is required for the inhibition of cytosolic LPS-induced pyroptosis in hepatocytes.

To investigate whether the cytosolic LPS accumulation is Caspase-11 dependent, we examined whether knockdown of Caspase-11 could impact LPS accumulation in hepatocytes. Knockdown of Caspase-11 by siRNA did not change the cytosolic FITC-LPS abundance in both WT and *Hspa12a*^*−/−*^ hepatocytes, respectively, compared with their scramble controls (Fig. S13a, b).

### HSPA12A deficiency exacerbates LPS-induced hepatic inflammation

**Fig. 4:**
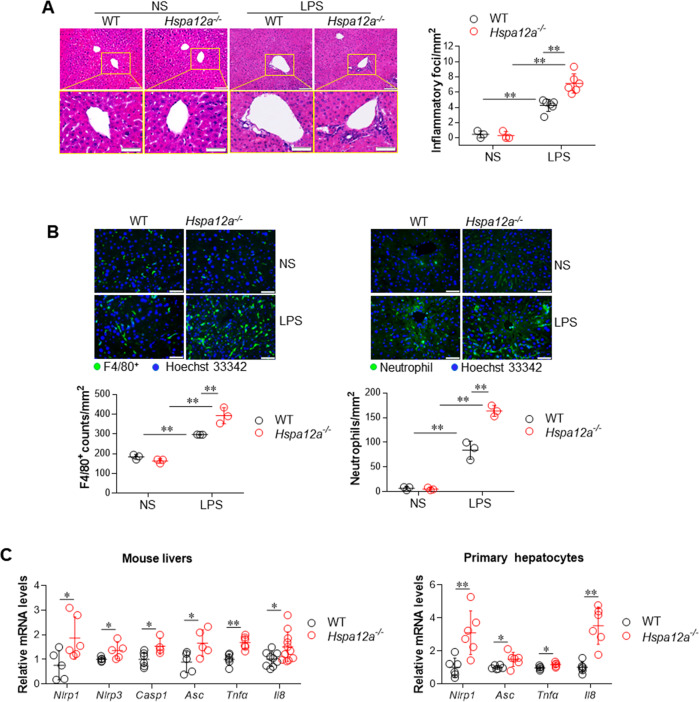
HSPA12A deficiency promoted the LPS-induced inflammatory response. Liver tissues were collected from mice 6 h after LPS or normal saline (NS) treatment. Also, cultured primary hepatocytes were collected after LPS incubation for 6 h. The following analysises were performed. **a** Inflammatory foci. H&E staining was performed on paraffin-embedded section of mouse livers. Inflammatory foci were observed and quantified using a microscope. Data are mean ± SD, **P < 0.01 by two-way ANOVA followed by Tukey’s test. *n* = 3/NS group and *n* = 6/LPS group. Scale bar = 100 μm (upper panels) and 50 μm (down panels). **b** Macrophage and neutrophil recruitments. Immunostaining for F4/80 (macrophage) and neutrophils was performed on frozen sections of mouse livers. Hoechst 33342 was counter stained to visualize nuclei. The staining was observed and quantified using a fluorescence microscope. Data are mean ± SD, **P < 0.01 by two-way ANOVA followed by Tukey’s test. *n* = 3/group. Scale bar = 50 μm. **c** Expression of mRNA levels. Levels of the indicated mRNA expression in livers (left panel) and primary hepatocytes (right panel) were evaluated using real-time PCR. Data are mean ± SD, *P < 0.05 and **P < 0.01 by Student’s two-tailed unpaired *t* test. *n* = 4–11/group.

Previous studies have demonstrated that pyroptosis causes inflammatory damage [5, 26, 27]. As shown in Fig. 4a, LPS administration increased the number of inflammatory foci in the livers of both genotypes, but there were still more inflammatory foci in *Hspa12a*^*−/−*^ livers than in WT livers. In addition, we found that the LPS-induced recruitment of macrophages (F4/80^+^) and neutrophils was more pronounced in *Hspa12a*^*−/−*^ livers than WT livers (Fig. 4b). In agreement with this, *Hspa12a*^*−/−*^ livers showed higher mRNA expression of proinflammatory mediators, including *Nlrp1*, *Nlrp3*, *Casp1*, *Asc*, *Tnfa*, and *Il8*, than WT livers following LPS challenge (Fig. 4c, left panel). Similar results were obtained in primary hepatocyte cultures, which showed higher mRNA levels of *Nlrp1*, *Asc*, *Tnfa*, and *Il8* in *Hspa12a*^*−/−*^ hepatocytes than in WT hepatocytes after LPS incubation (Fig. 4c, right panel).

### HSPA12A deficiency reduces the expression of AOAH, a lipase responsible for the inactivation of cytosolic LPS

**Fig. 5:**
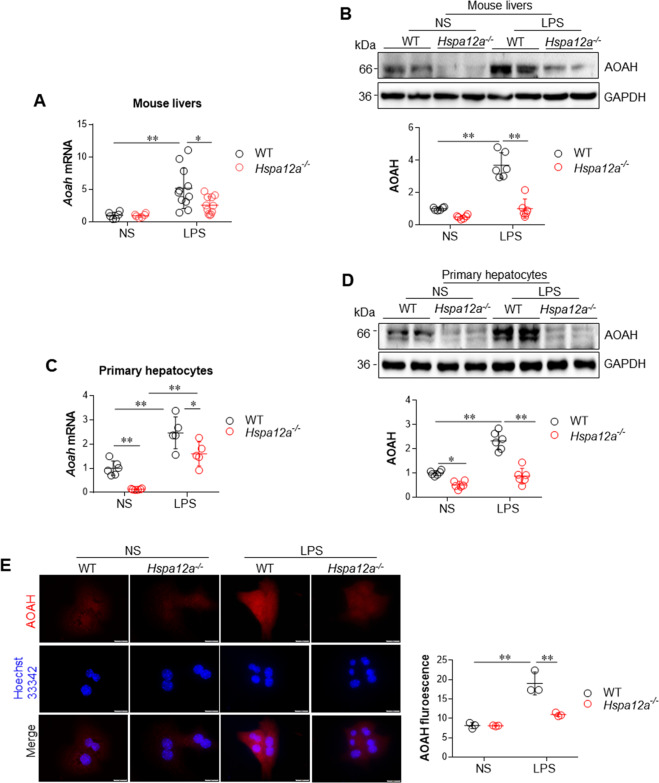
HSPA12A deficiency suppressed AOAH expression in both mouse livers in vivo and primary hepatocytes in vitro. Liver tissues were collected from mice 6 h after LPS or normal saline (NS) treatment. In another set of experiments, cultured primary hepatocytes were collected after LPS or NS incubation for 6 h. The following analysises were performed. **a** Liver *Aoah* mRNA expression was evaluated using real-time PCR. Data are mean ± SD, *P < 0.05 and ** *P* < 0.01 by two-way ANOVA followed by Tukey’s test. *n* = 6/NS group and *n* = 11/LPS group. **b** Liver AOAH protein expression was evaluated using immunoblotting. Data are mean ± SD, **P < 0.01 by two-way ANOVA followed by Tukey’s test. *n* = 6 /group. **c** Primary hepatocyte *Aoah* mRNA expression was evaluated using real-time PCR. Data are mean ± SD, *P < 0.05 and **P < 0.01 by two-way ANOVA followed by Tukey’s test. *n* = 6/NS group and *n* = 5/LPS group. **d** Primary hepatocyte AOAH protein expression was evaluated using immunoblotting. Data are mean ± SD, *P < 0.05 and **P < 0.01 by two-way ANOVA followed by Tukey’s test. *n* = 6/group. **e** AOAH protein was immunostained in primary hepatocytes. Hoechst 33342 was used to counter stain nuclei. Data was expressed as fluorescence intensity/cell. Scale bar = 20 μm. Data are mean ± SD, **P < 0.01 by Student’s two-tailed unpaired *t* test. *n* = 3/group.

To determine how HSPA12A deficiency increases cytosolic LPS accumulation and exacerbates hepatocyte pyroptosis, we measured AOAH expression in mouse livers because AOAH is a lipase responsible for the inactivation of LPS [10, 11]. Following LPS challenge, *Aoah* mRNA expression increased in WT livers but remained unchanged in *Hspa12a*^*−/−*^ livers when compared with their respective NS controls (Fig. 5a). In line with it, AOAH protein expression increased in WT livers but remained unchanged in *Hspa12a*^*−/−*^ livers when compared with their respective NS controls following LPS challenge (Fig. 5b).

Next, we determined whether HSPA12A deficiency in hepatocytes results in a reduction in AOAH expression. We found that the LPS-induced increase of *Aoah* mRNA expression was attenuated in *Hspa12a*^*−/−*^ hepatocytes compared with WT hepatocytes (Fig. 5c). Furthermore, AOAH protein expression was higher in WT hepatocytes, but remained unchanged in *Hspa12a*^*−/−*^ hepatocytes following LPS exposure, when compared with their respective NS controls (Fig. 5d). Notably, both immunoblotting and immunofluorescence demonstrated a lower AOAH protein content in *Hspa12a*^*−/−*^ hepatocytes than in WT hepatocytes in the presence of LPS (Fig. 5d, e). The data indicates that LPS was not as efficiently de-activated in the absence of HSPA12A.

### Overexpression of HSPA12A upregulates AOAH expression, reduces cytosolic LPS content, and inhibits pyroptosis in primary hepatocytes following LPS incubation

**Fig. 6:**
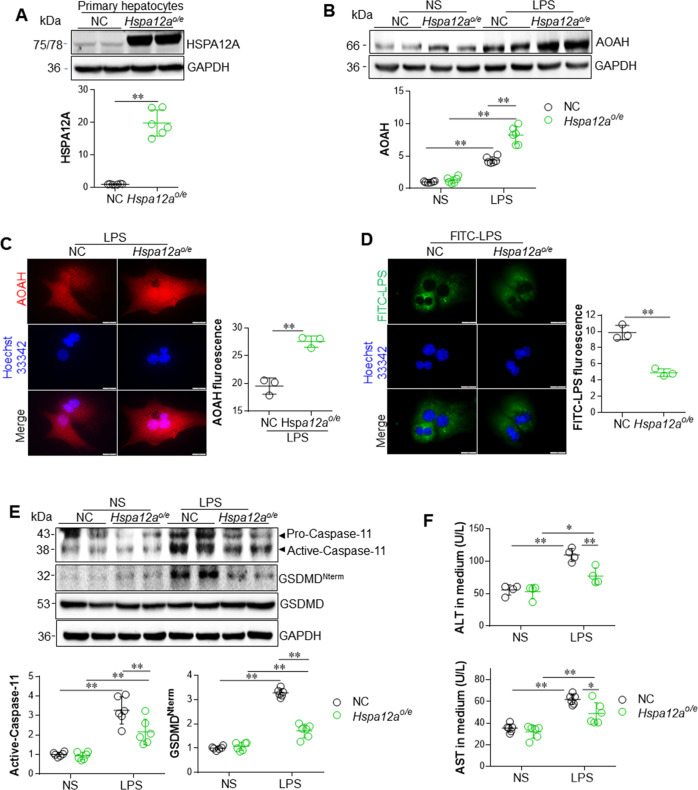
Overexpression of HSPA12A upregulated AOAH expression, reduced cytosolic LPS content, inhibited Caspase-11-mediated pyroptosis in primary hepatocytes upon LPS incubation. WT primary hepatocytes were infected with *Hspa12a*-adenovitus to overexpress HSPA12A (*Hspa12a*^*o/e*^). WT hepatocytes infected empty virus served as negative controls (NC). After incubation with LPS (or FITC-LPS) or normal saline (NS) for 6 h, the following analysises were performed. **a** Expression of HSPA12A was examined by immunoblotting. Data are mean ± SD, **P < 0.01 by Student’s two-tailed unpaired *t* test. *n* = 6/group. Endogenous HSPA12A was 75 kDa, whereas Exogenous HSPA12A was 78 kDa due to containing 3 flags. **b** AOAH expression was evaluated using immunoblotting. Data are mean ± SD, **P < 0.01 by two-way ANOVA followed by Tukey’s test. *n* = 6/group. **c** AOAH immunofluorescence was examined in LPS-incubated primary hepatocytes. Hoechst 33342 was used to counter stain nuclei. Data were expressed as fluorescence intensity/cell. Scale bar = 20 μm. Data are mean ± SD, **P < 0.01 by Student’s two-tailed unpaired *t* test. *n* = 3/group. **d** After incubation with FITC-LPS for 6 h, the primary hepatocytes were counter stained with Hoechst 33342. The stained fluorescence was observed and quantified using a fluorescence microscope. Data were expressed as FITC fluorescence intensity *10^3^/mm^2^ cell area. Scale bar = 20 μm. Data are mean ± SD, **P < 0.01 by Student’s two-tailed unpaired *t* test. *n* = 3/group. **e** Caspase-11 activation and GSDMD cleavage was examined using immunoblotting. Blots for GAPDH served as loading controls. Data are mean ± SD, **P < 0.01 by two-way ANOVA followed by Tukey’s test. *n* = 6/group. **f** ALT and AST activities in culture medium were analyzed. Data are mean ± SD, *P < 0.05 and **P < 0.01 by two-way ANOVA followed by Tukey’s test. *n* = 4/ALT group and *n* = 6/AST group.

Next, we determined whether overexpression of HSPA12A would ameliorate LPS-induced hepatocyte injury. To this end, HSPA12A was overexpressed (*Hspa12a*^*o/e*^) in WT primary hepatocytes by infection with *Hspa12a*-adenovirus, and cells infected with empty adenovirus served as negative controls (NC), as previously described [17] (Fig. 6a). In the presence of LPS, *Hspa12a*^*o/e*^ hepatocytes showed higher *Aoah* mRNA expression than NC controls (Fig. S14). Furthermore, the LPS-induced upregulation of AOAH protein was more marked in *Hspa12a*^*o/e*^ hepatocytes than NC controls (Fig. 6b). In addition, immunofluorescence showed greater AOAH protein abundance in *Hspa12a*^*o/e*^ hepatocytes than in NC cells following incubation with LPS (Fig. 6c).

Consistent with the upregulation of AOAH, less cytosolic LPS was present within *Hspa12a*^*o/e*^ primary hepatocytes than in NC cells following FITC-LPS incubation (Fig. 6d), suggesting that HSPA12A may inhibit the cytosolic LPS-induced pyroptosis of hepatocytes. Indeed, the LPS-induced Caspase-11 activation and GSDMD^Nterm^ peptide generation were attenuated in *Hspa12a*^*o/e*^ hepatocytes compared with NC cells (Fig. 6e). Accordingly, the LPS-induced ALT and AST leakage from primary hepatocytes was also attenuated by HSPA12A overexpression (Fig. 6f).

### The hepatoprotective effect of HSPA12A is mediated via upregulation of AOAH

**Fig. 7:**
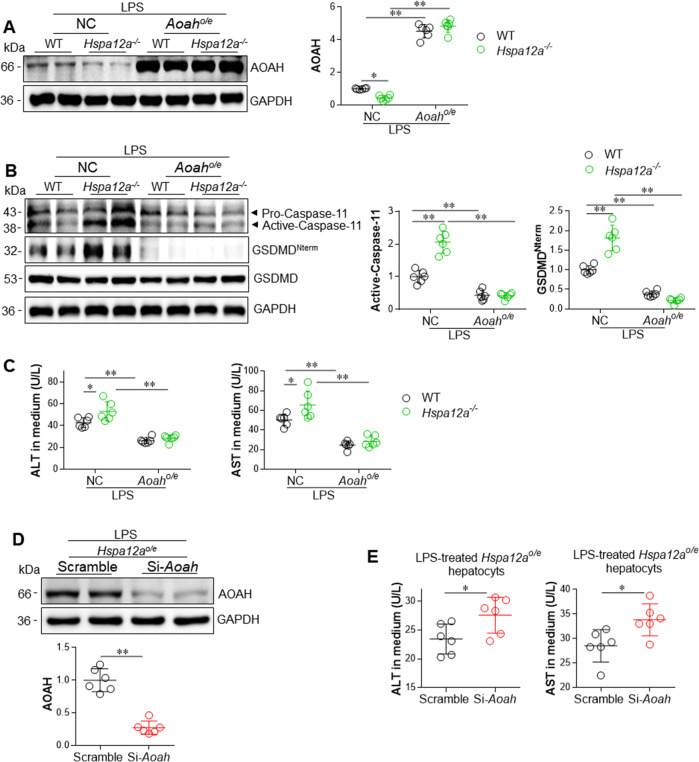
The hepatic protection of HSPA12A was mediated via upregulation of AOAH. Overexpression of AOAH reversed the promotion effect of HSPA12A deficiency in LPS-induced hepatocyte damage. WT and *Hspa12a*^*−/−*^ primary hepatocytes were infected with *Hspa12a*-adenovitus to overexpress AOAH (*Aoah*^*o/e*^), and *Hspa12a*^*−/−*^ hepatocytes infected empty virus served as negative controls (NC). Upon LPS incubation for 6 h, AOAH expression (**a**), Caspase-11 activation and GSDMD cleavage (**b**) were examined using immunoblotting. The activities of ALT and AST in culture medium were also measured (**c**). Data are mean ± SD, *P < 0.05 and **P < 0.01 by two-way ANOVA followed by Tukey’s test. *n* = 6/group. Knockdown of AOAH promoted hepatocyte injury in *Hspa12a*^*o/e*^ primary hepatocytes upon LPS incubation. AOAH was knockdown in *Hspa12a*^*o/e*^ primary hepatocytes by siRNA, and Scramble RNA-transfected *Hspa12a*^*o/e*^ hepatocytes cells served as controls. Upon LPS incubation for 6 h, AOAH expression was examined in cells using immunoblotting (**d**) and ALT and AST activities in culture medium were also measured (**e**). Data are mean ± SD, *P < 0.05 and **P < 0.01 by Student’s two-tailed unpaired *t* test. *n* = 6/group.

To determine whether the protection of HSPA12A against LPS-induced pyroptosis is mediated through upregulation of AOAH, we performed loss- and gain- of AOAH function experiments in primary hepatocytes. We first determined whether AOAH overexpression would rescue the promoted pyroptosis by HSPA12A deficiency. To this end, AOAH was overexpressed in WT and *Hspa12a*^*−/−*^ primary hepatocytes by infection with *Aoah*-adenovirus, and the hepatocytes infected with empty adenovirus served as NC controls (Figs. 7a, S15). We found that AOAH overexpression prevented the HSPA12A deficiency-induced increases in Caspase-11 activation and GSDMD^Nterm^ generation following LPS incubation (Fig. 7b). In addition, AOAH overexpression prevented the HSPA12A deficiency-induced increases in ALT and AST leakage from LPS-treated primary hepatocytes (Fig. 7c). In striking contrast, knockdown of AOAH in *Hspa12a*^*o/e*^ primary hepatocytes, which was achieved by transfection with *Aoah*-targeting siRNA (Fig. 7d), increased ALT and AST leakage from hepatocytes compared with that from scrambled siRNA-transfected *Hspa12a*^*o/e*^ hepatocytes following LPS exposure (Fig. 7e). Taken together, these findings suggest that the HSPA12A-induced protection against LPS-induced hepatocyte pyroptosis is *via* the upregulation of AOAH.

### HSPA12A upregulates AOAH expression through an interaction with PGC-1α

**Fig. 8:**
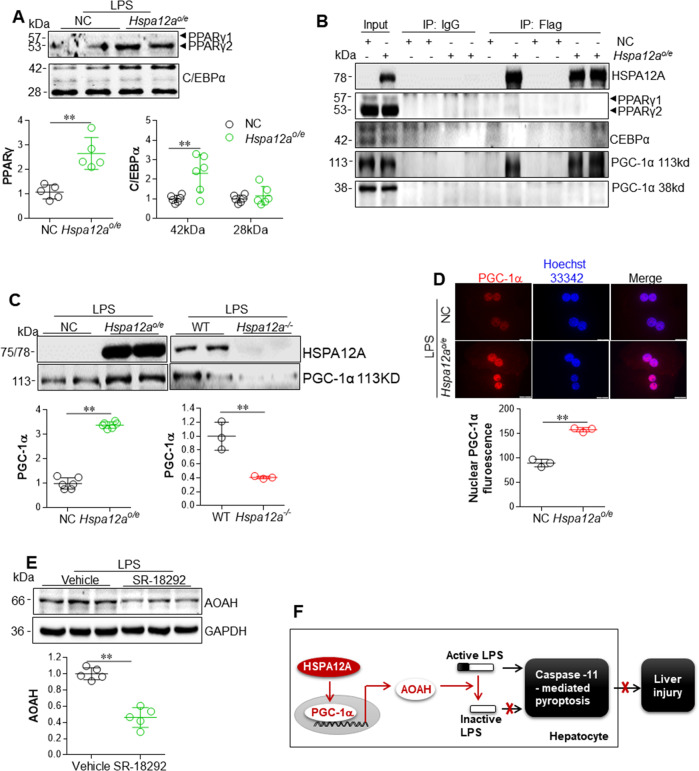
HSPA12A upregulated AOAH expression through interaction with PGC-1α in hepatocytes. **a** HSPA12A increased nuclear contents of PPARγ and C/EBPα in LPS-treated hepatocytes. WT primary hepatocytes were infected with *Hspa12a*-adenovitus to overexpress HSPA12A (*Hspa12a*^*o/e*^). WT hepatocytes infected with empty virus served as negative controls (NC). After incubation with LPS for 6 h, the nuclear fractions were prepared for immunoblotting with the indicated antibodies. Data are mean ± SD, **P < 0.01 by Student’s two-tailed unpaired *t* test. *n* = 5 for PPARγ group, *n* = 6 for C/EBPα group. **b** Interaction between HSPA12A and PGC-1α in hepatocytes. Primary WT hepatocytes that overexpressing the flag-tagged HSPA12A (*Hspa12a*^*o/e*^) were incubated with LPS for 6 h. Primary hepatocytes infected empty virus served as negative controls (NC). Cellular protein extracts were immunoprecipitated with primary antibody for flag. The immunoprecipitates were blotted with the indicated antibodies. Protein extracts without immunoprecipitation (input) served as positive controls, and immunoprecipitates from IgG incubation served as negative controls. Note that only PGC-1α was recovered in flag-tagged HSPA12A immunoprecipitates. **c** Nuclear PGC-1α content was increased by HSPA12A overexpression but decreased by HSPA12A deficiency. *Hspa12a*^*o/e*^ primary hepatocytes and its NC controls, *Hspa12a*^−/−^ primary hepatocytes and its WT controls were treated with LPS for 6 h. Nuclear fractions were prepared for immunoblotting against PGC-1α and HSPA12A. Data are mean ± SD, **P < 0.01 by Student’s two-tailed unpaired *t* test. *n* = 6/group for left panels and *n* = 3/group for right panels. **d** HSPA12A increased nuclear PGC-1α abundance in LPS-treated hepatocytes. *Hspa12a*^*o/e*^ and NC primary hepatocytes were treated with LPS for 6 h. PGC-1α in nuclei was examined by immunostaining. Hoechst 33342 was used to counter staining nuclei. Data were expressed as fluorescence intensity/nucleus. Scale bar = 20 μm. Data are mean ± SD, **P < 0.01 by Student’s two-tailed unpaired *t* test. *n* = 3/group. **e** Inhibition of PGC-1α decreased AOAH expression in *Hspa12a*^***o/e***^ hepatocytes. *Hspa12a*^*o/e*^ primary hepatocytes were treated with PGC-1α inhibitor SR-18292 or vehicle for 12 h followed by incubation with LPS for 6 h. AOAH expression was examined by immunoblotting. Data are mean ± SD, **P < 0.01 by Student’s two-tailed unpaired *t* test. *n* = 5/group. **f** Mechanism scheme. By directly binding to PGC-1α, HSPA12A promotes PGC-1α translocation to nuclei, thereby promotes AOAH expression for LPS inactivation, and ultimately leads to hepatic protection through inhibition of Caspaase11-mediated pyroptosis of hepatocyte.

Finally, we sought to answer the question about how HSPA12A regulates AOAH expression. Because HSPA12A demonstrated nuclear translocation following LPS exposure (Fig. 1b), we hypothesized that it may bind and promote the nuclear translocation of certain transcription factors or transcriptional coactivators that are required for *Aoah* gene expression. Gene promoter analysis (http://gene-regulation.com) showed the presence of putative binding sites of CCAAT/enhancer-binding protein alpha (C/EBPα) and peroxisome proliferator- activated receptor gamma (PPARγ), two transcription factors, within the *Aoah* promoter. Indeed, we observed a higher abundance of C/EBPα and PPARγ proteins in the nuclear fractions of *Hspa12a*^*o/e*^ primary hepatocytes following LPS incubation (Fig. 8a). However, immunoprecipitation-immunoblotting analysis revealed that neither C/EBPα nor PPARγ proteins could be recovered from flag-tagged HSPA12A immunocomplexes prepared from LPS-treated primary hepatocytes (Fig. 8b), suggest no direct interaction of HSPA12A with C/EBPα or PPARγ.

Unexpectedly, PGC-1α of 113 kDa in size, but not of 38 kDa, was recovered from the flag-tagged HSPA12A immunocomplexes from LPS-incubated primary hepatocytes (Fig. 8b). Interestingly, the PGC-1α protein levels in nuclear fractions of primary hepatocytes were increased by HSPA12A overexpression but decreased in HSPA12A deficiency in the presence of LPS (Fig. 8c). Consistent with this, *Hspa12a*^*o/e*^ primary hepatocyte nuclei contained more PGC-1α protein than NC controls following LPS exposure (Fig. 8d). Importantly, treatment with SR-18292, a selective inhibitor of PGC-1α transcriptional activity [28], reduced AOAH expression in LPS-treated *Hspa12a*^*o/e*^ primary hepatocytes (Fig. 8e).

## Discussion

The present study has revealed that HSPA12A directs LPS inactivation to reduce susceptibility to pyroptosis of hepatocytes. This effect of HSPA12A is mediated through inhibition of cytosolic LPS-induced pyroptosis via PGC-1α-mediated AOAH expression in hepatocytes.

Among the superfamily of heat shock proteins, HSP70 and HSP90 are involved in liver injury during sepsis/septic shock [29–31]. As examples, inhibition of HSP90 reduces proinflammatory cytokine production and prevents LPS-induced liver injury [29], whereas induction of endogenous HSP70 and HSP27 or treatment with exogenous HSP70 ameliorates the hepatic and cardiac lesions associated with the anti-inflammatory responses during experimental septic shock [30–32]. In the present study, we found that HSPA12A, a novel member of HSP70 family, translocates to the nucleus of hepatocytes following LPS exposure, suggesting its possible involvement in the hepatic injury during sepsis. Indeed, we found that LPS-induced hepatic injury was exacerbated by HSPA12A deficiency in mice. Moreover, the primary hepatocyte injury induced by LPS was exacerbated by HSPA12A deficiency but attenuated by HSPA12A overexpression. We also found lower body temperature and activity in HSAP12A knockout mice than WT controls after LPS challenge. Taken together, these results provide clear evidence that HSPA12A protects the liver from LPS-induced injury.

LPS enters the circulation in large quantities following Gram-negative bacterial infection and is a pathogen-associated molecular pattern. This circulating LPS activates canonical mammalian host-cell detection mechanisms by binding to cell-surface TLR4. However, recent studies conducted in macrophages and endothelial cells have shown that another intracellular/cytosolic LPS-sensing pathway may be more important for the development of sepsis/septic shock [4, 5]. Intracellular LPS, which is generated by Gram-negative bacteria within cells or imported by a mechanism involving lipoproteins and high mobility group box 1 [8, 33], is sensed by proinflammatory Caspase-4 and -5 in humans or their ortholog Caspase-11 in rodents and leading to the generation of GSDMD^Nterm^, which induces pyroptosis by forming membrane pores and increasing the secretion of proinflammatory mediators [8]. Pyroptosis is a form of programmed lytic cell death that is induced by proinflammatory Caspases, but little is known about its role in hepatocyte death and acute liver injury. In the present study, we have made three key findings. First, intracellular LPS was present in both hepatocytes of livers of LPS-treated mice and in primary hepatocyte cultures incubated with LPS. Second, LPS induced pyroptosis in both mouse livers and primary hepatocyte cultures, as demonstrated by Caspase-11 activation and GSDMD^Nterm^ generation (markers of pyroptosis). Third, intracellular LPS accumulation and Caspase-11-mediated hepatocyte pyroptosis were promoted by HSPA12A deficiency but attenuated by HSPA12A overexpression. These findings imply that LPS can enter hepatocytes and activate Caspase-11-mediated pyroptosis, and that this is inhibited by HSPA12A overexpression.

Evidence has shown that Gram-negative enteropathogens activate TLR4-TRAM for type I interferon induction through the interferon regulatory factors IRF3/7, while Type I interferon can stimulate Caspase-11 and leading to inflammatory tissue damage during endotoxic shock [9, 34]. However, the sensing of intracellular LPS by Caspase-4/5/11 is critical for the activation of pyroptosis. This raises the possibility that the inactivation of intracellular LPS could be protective against the LPS-induced cell damage. Indeed, previous studies have demonstrated that LPS can be cleared effectively by hepatocytes and Kupffer cells followed by excretion via the bile canalicular system [35, 36]. Recent studies have shown that intracellular LPS can be biologically inactivated by AOAH, which results in a desensitization of LPS by Caspase-4/5/11 [10, 21]. AOAH is expressed in various phagocytes, including Kupffer cells and hepatic dendritic cells; however, it was unknown whether AOAH is expressed in hepatocytes. In the present study, we have shown that AOAH was expressed in hepatocytes and was upregulated in response to LPS exposure. Moreover, HSPA12A overexpression increased, but HSPA12A deficiency reduced, AOAH expression in hepatocytes. Importantly, overexpression AOAH prevented the HSPA12A deficiency-induced promotion of the LPS-induced, Caspase-11-mediated pyroptosis of hepatocytes. When taken into account that *Hspa12a*^*−/−*^ mice exhibited exacerbated liver injury, lower body temperature and less activities, our findings suggest that HSPA12A protects liver and improves systemic conditions from LPS challenge via an AOAH-dependent mechanism.

To further explore the mechanism whereby HSPA12A upregulates AOAH expression, we aimed to identify the transcription factors responsible for regulating AOAH gene expression. Gene promoter analysis (http://gene-regulation.com) demonstrated putative binding sites for C/EBPα and PPARγ within the *Aoah* promoter. Considering this finding alongside the fact that HSPA12A translocated to the nucleus following LPS challenge, we hypothesized that HSPA12A may bind and promote the nuclear translocation of these transcription factors, resulting in transcription of *Aoah* gene. However, although we identified an effect of HSPA12A on the nuclear translocation of C/EBPα and PPARγ in hepatocytes, no direct interaction of HSPA12A with C/EBPα or PPARγ was detected using immunoprecipitation- immunoblotting analysis. However, this analysis did reveal that HSPA12A forms a complex with PGC-1α in hepatocytes, and that HSPA12A overexpression increased the nuclear abundance of PGC-1α. Furthermore, inhibition of the transcriptional activity of PGC-1α using SR-18292 [28] prevents HSPA12A-induced AOAH expression in hepatocytes. Collectively, these results indicate that HSPA12A interacts with PGC-1α and increases its nuclear translocation, thereby inducing AOAH expression for cytosolic LPS inactivation, which ultimately leading to inhibition of the Caspase-11-mediated pyroptosis of hepatocytes that manifested reduced liver injury and improved systemic conditions during endotoxemia (Fig. 8f).

In conclusion, we have shown that HSPA12A protects mouse livers from the LPS-induced experimental sepsis. This action of HSPA12A is achieved through inhibition of intracellular LPS-induced pyroptosis, which is dependent on PGC-1α-mediated AOAH expression. These findings suggest that hepatocyte HSPA12A represents a viable target for the management of liver injury in sepsis patients.

## Materials and methods

### Reagents

*Escherichia coli* LPS (0111:B4), FITC-conjugated LPS from *E. coli* 0111:B4 (FITC-LPS) and paraformaldehyde (PFA) was from Sigma-Aldrich (St. Louis, MO). Collagenase Type 4 was from Worthington biochemical Corporation (Lakewood, NJ). Percoll density gradient media was from GE Healthcare (Uppsala, Sweden). Normal goat serum was from Jackson ImmunoResearch (West Grove, PA). Trizol reagent was from Life Technology (Carlsbad, CA). SYBR Green Master and bovine serum albumin (BSA) was from Roche (Basel, Switzerland). DMEM medium and fetal bovine serum (FBS) was from Gibco (Shelton, CT). High-sig ECL western blotting substrate was from Tanon (Shanghai, China). Protein A-Agarose was from Santa Cruz Biotechnology (Dallas, TX). Lactic acid dehydrogenase (LDH) assay kit was from Jiancheng Biotech (Nanjing, China).

### Animals

Conditional *Hspa12a* knockout mice were generated using the *lox*P and *Cre* recombinant system as described in our previous studies [16, 17]. The mice were bred at the Model Animal Research Center of Nanjing University and were maintained in the Animal Laboratory Resource Facility of the same institution. All experiments conformed to the Guide for the Care and Use of Laboratory Animals published by the US National Institutes of Health (NIH Publication, 8th Edition, 2011). The animal care and experimental protocols were approved by Nanjing University’s Committee on Animal Care. All experiments conformed to international guidelines on the ethical use of animals.

Mice (C57BL/6 background) were randomly assigned to all analyses. Investigators were blinded to the histological analysis. Investigators involved in animal handling, sampling, and raw data collection were not blinded.

### LPS treatment

An LPS-induced experimental sepsis model was established by injection male mice (8–10 weeks of age) intraperitoneally with LPS or FITC-LPS at a dosage of 5 mg/kg body weight. NS-treated mice served as controls. Food and water were provided ad libitum. Six hours after LPS treatment, blood was sampled and tissues were collected for the indicated measurements.

In primary hepatocyte experiments, cells were incubated with LPS or FITC-LPS (500 ng/ml) for 6 h. Saline-treated primary hepatocytes served as controls. Culture medium and cells were collected for the indicated analysises.

### Immunoblotting and immunoprecipitation-immunoblotting

Cytosolic and pellet fractions were prepared from livers or cells. Western blotting was performed according to our previous methods [16, 17]. To control for lane loading, the membranes were probed with anti-GAPDH antibody for cytosolic proteins and anti-Lamin A/C antibody for pellet proteins.

For analyzing interaction between HSPA12A and other proteins by immunoprecipitation- immunoblotting, WT primary hepatocytes were overexpressed with flag-tagged HSPA12A. After challenged with LPS for 6 h, cells were collected for protein extraction. Aliquots of equal volume and protein content were precipitated with anti-flag antibodies, followed by Western blotting for C/EBPα, PPARγ, PGC-1α and HSPA12A as described previously [17, 37].

The primary antibodies used in the experiments are listed in Supplementary Table S1.

### Evaluation of LPS abundance within hepatocytes

In mouse experiments, serum and tissues of livers were collected 6 h after FITC-LPS treatment. The LPS abundance in serum and liver homogenates was indicated by FITC fluorescence intensity using a fluorometer (Synergy HT, BIO-TEK, USA) at excitation /emission wavelengths of 490/530 nm. For visualizing the FITC-LPS in hepatocytes, the frozen sections of PFA-fixed liver tissues were immunostained with alpha-fetoprotein (AFP). Hoechst33342 was used to counter stain nuclei.

In primary hepatocyte experiments, cells were fixed with PFA after incubation with FITC-LPS for 6 h. The fixed cells were counter stained with Hoechst33342 to visualize nuclei. The staining was observed using a fluorescence microscope and quantified using Cellsens Dimention 1.15 software (Olympus, Tokyo, Japan).

### Quantitative real-time PCR

Quantitative real-time PCR was performed as described previously [16, 17]. Briefly, Total RNA was isolated for cDNA synthesis. The expressions of indicated genes were estimated by real-time PCR using the SYBR Green Master. The PCR results of *Actin* served as internal controls. The primers used for PCR are listed in Supplementary Table S2.

### Biochemical analysis

Activities of aspartate aminotransferase (AST) and alanine aminotransferase (ALT) in mouse serum and cell culture medium were measured using a Beckman Coulter AU5800 Chemistry System analyzer (Brea, CA).

### Histological analysis and immunofluorescence staining

Paraffin-embedded liver sections were stained with H&E to evaluate the inflammation foci formation. Immunofluorescence staining on frozen liver sections or primary hepatocytes was performed as described previously using appropriate antibodies [16, 17] (Supplementary Table S1). Briefly, after incubation with the indicated primary antibodies (1:100) overnight at 4 °C, Cy3- or FITC-conjugated appropriate secondary antibodies were added to the sections to visualize the staining. Hoechst 33342 reagent was used to counterstain the nuclei. The staining was observed and quantified in ten randomly selected ten areas of each sample using a fluorescence microscope with Cellsens Dimention 1.15 software (Olympus, Tokyo, Japan).

### Adenovirus construction

The adenoviral vector containing a three flags-tagged coding region of mouse *Hspa12*a (NM_175199) was generated by GeneChem (Shanghai, China) as described in our previous studies [16, 17]. The adenoviral vector containing a three flags-tagged coding region of mouse *Aoah* (NM_012054) was generated by GeneChem (Shanghai, China). A schematic overview of *Aoah* virus construction is shown in Supplementary Fig. S4.

### Primary hepatocyte isolation and culture

Primary hepatocytes were isolated and grown according to our previous methods [17]. Briefly, hepatocytes were isolated from 7–10-week-old mice by digestion with 0.06% collagenase type IV, followed by centrifugation on a 25–50% Percoll gradient. Primary hepatocytes were grown in DMEM supplemented with 10% FBS and 0.01 mM dexamethasone.

To overexpress HSPA12A (*Hspa12a*^*o/e*^) or AOAH (*Aoah*^*o/e*^), primary hepatocytes were infected with adenovirus carrying the expression sequence of *Hspa12a* or *Aoah* for 48 h. Cells infected with empty adenovirus served as normal expression controls (NC).

To knockdown of AOAH or Caspase-11 expression, primary hepatocytes were transfected with siRNA that targeting mouse *Aoah* mRNA (Si-*Aoah*) or *Caspase-11* mRNA for 48 h, respectively. The primary hepatocytes transfected with Scramble RNA served as controls (Scramble). The siRNA sequences were shown in Supplementary Table S3.

### Statistical analysis

Data are expressed as the mean ± standard deviation. Groups were compared using Student’s two-tailed unpaired *t* test, or using two-way ANOVA followed by Tukey’s post-hoc test. A *P* value of *<* 0.05 was considered significant.

## Supplementary information

supplemental materials

Supplementary Figure S1

Supplementary Figure S2

Supplementary Figure S3

Supplementary Figure S4

Supplementary Figure S5

Supplementary Figure S6

Supplementary Figure S7

Supplementary Figure S8

Supplementary Figure S9

Supplementary Figure S10

Supplementary Figure S11

Supplementary Figure S12

Supplementary Figure S13

Supplementary Figure S14

Supplementary Figure S15

supplemental tables
