# Supplementary material for: HSPA12A attenuates lipopolysaccharide-induced liver injury through inhibiting caspase-11-mediated hepatocyte pyroptosis via PGC-1α-dependent acyloxyacyl hydrolase expression
Source: Cell Death Differ. 2020 Apr 24;27(9):2651–67. doi: 10.1038/s41418-020-0536-x (PMC7429872; doi:10.1038/s41418-020-0536-x)
Supplement: Supplementary file 1 — supplemental materials [file 41418_2020_536_MOESM1_ESM.pdf]

Supplementary Materials

1. Methods

1.1 Dose-effects and time-course of LPS on hepatic HSPA12A expression and injury in mice.

Mice were treated with LPS for 6 h at the dosages of 2.5, 5, 10 and 20 mg/kg by intraperitoneally injection. In another set of experiments, mice were treated with 5 mg/kg LPS by intraperitoneally injection for 3, 6, 12 and 24 h. Normal saline (NS)-treated mice were served as controls. At the end of experiments, serum was collected for measurements of alanine transaminase (ALT) and aspartate transaminase (AST) activities, and livers were collected for the examination of HSPA12A expression and Caspase-11 activation.

1.2 Dose-effects and time-course of LPS on HSPA12A expression and injury in primary hepatocytes.

Primary hepatocytes were incubated with LPS for 6 h at the dosages of 250, 500, 1000 and 2000 ng/ml. In another set of experiments, primary hepatocytes were incubated with 500 ng/ml LPS for 3, 6, 12 and 24 h. NS-treated hepatocytes served as controls. At the end of experiments, culture medium was collected for measurements

of ALT and AST activities, and hepatocytes were collected for the examination of HSPA12A expression and Caspase-11 activation.

1.3 Body temperature and animal activities

Body temperature in mice was detected at 2, 4 and 6 h following LPS (5 mg/kg) treatment using a rectal probe. Animal activities were also evaluated at 2, 4 and 6 h following LPS (5 mg/kg) treatment according to previous methods [1]. The measurements before LPS treatment served as baseline controls. In brief, the scoring system of activity includes two principal tasks: hunched posture and spontaneous rapid movements interspersed with eating and drinking. The scoring system ranged from 0 to 4, in which 4 (normal) denotes that mice intersperse movement spontaneously and rapidly with eating and drinking but without hunched posture, and 0 (severe) is continual hunched posture without movement. The investigator was blinded to the treatment.

1.4 **Measurement of Blood pressure, blood gas, blood creatinine (Cr) and urea-nitrogen (Urea)** ***Noninvasive blood pressure.***

Six hours after LPS (5 mg/kg) or NS treatment, mouse systolic blood pressure (SBP) was measured using a non-invasive tail cuff computerized system (ACL-NIBP, Alcott Biotech, China) as described in our previous study [2]. All the measured mice were pre-trained for 5 consecutive days in the pre-warmed tail-cuff device to

accustom them to the procedure. The investigator was blinded to the treatment.

Blood gas analysis. Six hours after LPS (5 mg/kg) or NS treatment, mice were anesthetized (1.5% isofluorane), intubated and mechanically ventilated. Arterial blood was drawn from the left ventricle for blood gas measurements, including blood oxygen saturation (SO_2), partial pressure of blood oxygen (pO_2), and partial pressure of blood carbon dioxide pressure (pCO_2) using iSTAT Analyzer MN:300 (Abbott Park, IL).

Blood creatinine (Cr) and urea-nitrogen (Urea). Six hours after LPS (5 mg/kg) or NS treatment, serum was separated for the analyses of Cr and Urea using a Beckman Coulter AU5800 Chemistry System analyzer (Brea, CA).

1.5 Animal mortality

Mice were checked every 30 min within 6 h after LPS (5 mg/kg) treatment. The investigator was blinded to the treatment.

1.6 Histological examination of spleen and intestine

Six hours after LPS (5 mg/kg) or NS treatment, spleens and intestines were collected for paraffin-embedded sections. Hematoxylin and Eosin (H&E) staining was performed subsequently according to previous methods [3, 4, 5, 6].

1.7 Time-course of LPS accumulation in primary hepatocytes

Primary hepatocytes were incubated with FITC-LPS (500 ng/ml) for 3, 6, 12, and 24 h. At the end of experiments, cells were collected for Hoechst33342 staining to indicate nuclei. The stained fluorescence was observed and quantified using a fluorescence microscope. Data was expressed as FITC fluorescence intensity*$10^3/mm^2$ cell area.

1.8 Lactic acid dehydrogenase (LDH) leakage from LPS-treated primary hepatocytes

Primary hepatocytes were incubated with LPS (500 ng/ml) for 6 h. At the end of experiments, culture medium was collected for LDH activity analysis using the LDH assay kit according to our previous methods [7].

2. Figure legends

Figure S1. Dose-effect and time-course of LPS on HSPA12A expression in mouse livers *in vivo* and primary hepatocytes *in vitro.*

Mice (A) or primary hepatocytes (C) were treated with LPS for 6 h at the indicated concentrations. In other sets of experiments, mice were treated with 5 mg/kg LPS (B) or primary hepatocytes were incubated with 500 ng/ml LPS (D) for the indicated durations. Mouse livers or primary hepatocytes were collected for HSPA12A expression analysis by immunoblotting. Blots for GAPDH or Lamin A/C served as loading controls. Data are mean ± SD, ** $P < 0.01$ and * $P < 0.05$ vs. NS control by one-way ANOVA followed by Tukey's test. n =3/group.

Figure S2. *Hspa12a* mRNA expression in hepatocytes.

Hspa12a mRNA expression was examined in primary hepatocytes after incubation with LPS or NS for 6 h using real-time PCR. Data are mean ± SD. n = 6/group.

Figure S3. Body temperature, activity, SBP, blood gas, blood Urea and Cr, and mortality of mice.

Mice were treated with LPS (5 mg/kg) for 6 h. The indicated measurements were performed. Data are mean ± SD. ** $P < 0.01$ vs. basal control by one-way ANOVA

followed by Tukey's test (A, B), or ** $P < 0.01$ and * $P < 0.05$ by Student's two-tailed unpaired t test (C-E). Survival rate was analyzed by log−rank test (F). n = 6/group (A, B), n = 4/group (C), n = 3/group (D), n = 8-14/group (E), and n = 10/group (F).

Figure S4. Dose-effect and time-course of LPS on liver injury.

A. Mice were treated with LPS for 6 h at the indicated concentrations. Livers were collected for Caspase-11 activation using immunoblotting.

B. Mice were treated with 5 mg/kg LPS for the indicated durations. Livers were collected for Caspase-11 activation using immunoblotting.

C. Mice were treated with LPS for 6 h at the indicated concentrations. Serum was collected for ALT and AST activity analysis.

D. Mice were treated with 5 mg/kg LPS for the indicated durations. Serum was collected for ALT and AST activity analysis.

Data are mean ± SD, ** $P < 0.01$ vs. NS control by one-way ANOVA followed by Tukey's test. n =3/group (A, B) and n = 4/group (C, D).

Figure S5. Dose-effect and time-course of LPS on primary hepatocyte injury.

A. Primary hepatocytes were treated with LPS for 6 h at the indicated concentrations. Caspase-11 activation was examined using immunoblotting.

B. Primary hepatocytes were treated with 500 ng/ml LPS for the indicated durations. Caspase-11 activation was examined using immunoblotting.

C. Primary hepatocytes were treated with LPS for 6 h at the indicated concentrations. Culture medium was collected for ALT and AST activity analysis.

D. Primary hepatocytes were treated with 500 ng/ml LPS for the indicated durations. Culture medium was collected for ALT and AST activity analysis.

Data are mean ± SD, ** $P < 0.01$ and * $P < 0.05$ vs. NS control by one-way ANOVA followed by Tukey's test. n = 3/group (A, B), n = 6/group (C), n = 4-6/group (D).

Figure S6. Serum ALT and AST activities.

Mice were treated with 5 mg/kg LPS for the 6 h (n = 10/group) and 24 h (n = 3/group), respectively. Serum was collected for ALT and AST activity analysis. Data are mean ± SD, ** $P < 0.01$ and * $P < 0.05$ by two-way ANOVA followed by Tukey's test.

Figure S7. Body temperature and animal activities.

Mice were treated with 5 mg/kg LPS 6 h. Body temperature and animal activities were measured. Data are mean ± SD, ** $P < 0.01$ and * $P < 0.05$ vs. the time matched-*Hspa12a*$^{-/-}$ mice by two-way ANOVA followed by Tukey's test. n = 6/group.

Figure S8. Organ examination following LPS treatment.

Mice were treated with 5 mg/kg LPS 6 h. Blood gas (A, n = 3-5/group), Urea and Cr (B, n = 8-14/group) was analyzed. Also, histology of spleen (C, n = 3/group) and

intestine (D, n = 3/group) was examined by H&E staining on paraffin-embedded sections. Data are mean ± SD, ** P < 0.01 and * P < 0.05 by two-way ANOVA followed by Tukey's test. Scale bar = 100 μM.

Figure S9. HSPA12A deficiency increased LPS contents in serum of mice.

Six-hours after FITC-LPS treatment, serum were collected from mice. LPS abundance in serum was indicated by the FITC fluorescence intensity (arbitrary unites) that measured by a fluorometer at excitation/emission wavelengths of 490/530 nm. Data are mean ± SD, ** P < 0.01 by Student's two-tailed unpaired t test. n = 5/group.

Figure S10. Time-course of LPS accumulation in primary hepatocytes.

After incubation with FITC-LPS (500 ng/ml) for the indicated durations, the primary hepatocytes were counter stained with Hoechst33342. The staining was observed and quantified using a fluorescence microscope. Data was expressed as FITC fluorescence intensity $*10^3/mm^2$ cell area. Data are mean ± SD, ** P < 0.01 or * P < 0.05 vs. 3 h control by one-way ANOVA followed by Tukey's test. n =3/group. Scale bar = 20 μM.

Figure S11. LPS and vesicle staining.

After incubation with FITC-LPS (500 ng/ml) for 6 h, the primary hepatocytes were immunofluorescence stained for a vesicle marker Flotinllin-1. Hoechst33342 was used to counterstain nuclei. The staining was observed using a fluorescence

microscope. n =3/group. Scale bar = 10 μM.

Figure S12. LDH activity in culture medium.

After incubation with LPS or NS for 6 h, the medium of primary hepatocyte cultures were collected for LDH activity assay. Data are mean ± SD, ** $P < 0.01$ or * $P < 0.05$ by two-way ANOVA followed by Tukey's test. n =3/group.

Figure S13. Effects of Caspase-11 knockdown on cytosolic LPS accumulation.

Caspase-11 in primary hepatocytes was knocked-down by si-RNA (A). After incubation with FITC-LPS for 6, the primary hepatocytes were counter stained with Hoechst33342. The staining was observed and quantified using a fluorescence microscope. Data was expressed as FITC fluorescence intensity $*10^3/mm^2$ cell area. Data are mean ± SD, ** $P < 0.01$ by Student's two-tailed unpaired *t* test (A) or two-way ANOVA followed by Tukey's test (B). n = 3/group. NS., no significance. Scale bar = 20 μM.

Figure S14. *Aoah* mRNA expression.

WT primary hepatocytes were infected with *Hspa12a*-adenovitus to overexpress HSPA12A ($Hspa12a^{o/e}$). WT hepatocytes infected empty virus served as negative controls (NC). After incubation with LPS for 6 h, *Aoah* mRNA was evaluated using real-time PCR. Data are mean ± SD, * $P < 0.05$ by Student's two-tailed unpaired *t* test. n = 6 for WT group and n =5 for $Hspa12a^{o/e}$ group.

Figure S15. Schematic represents construction of adenovirus containing mouse AOAH expression sequence.

Full length of mouse *Aoah* CDS was inserted in the multiple clonal sites (MCS).

3. References

1. Walley KR, Thain KR, Russell JA, Reilly MP, Meyer NJ, Ferguson JF*, et al.* PCSK9 is a critical regulator of the innate immune response and septic shock outcome. *Sci Transl Med* 2014, **6**(258)**:** 258ra143.

2. Zhou H, Qian J, Li C, Li J, Zhang X, Ding Z*, et al.* Attenuation of cardiac dysfunction by HSPA12B in endotoxin-induced sepsis in mice through a PI3K-dependent mechanism. *Cardiovasc Res* 2011, **89**(1)**:** 109-118.

3. Nezic L, Amidzic L, Skrbic R, Gajanin R, Nepovimova E, Valis M*, et al.* Simvastatin Inhibits Endotoxin-Induced Apoptosis in Liver and Spleen Through Up-Regulation of Survivin/NF-kappaB/p65 Expression. *Front Pharmacol* 2019, **10:** 54.

4. Arima H, Hirate H, Sugiura T, Suzuki S, Takahashi S, Sobue K. IV injection of polystyrene beads for mouse model of sepsis causes severe glomerular injury. *J Intensive Care* 2014, **2**(1)**:** 21.

5. Guo S, Nighot M, Al-Sadi R, Alhmoud T, Nighot P, Ma TY. Lipopolysaccharide Regulation of Intestinal Tight Junction Permeability Is Mediated by TLR4 Signal Transduction Pathway Activation of FAK and MyD88. *J Immunol* 2015, **195**(10)**:** 4999-5010.

6. Gunther C, Buchen B, He GW, Hornef M, Torow N, Neumann H*, et al.* Caspase-8 controls the gut response to microbial challenges by Tnf-alpha-dependent and independent pathways. *Gut* 2015, **64**(4)**:** 601-610.

7. Ma R, Wang X, Lu C, Li C, Cheng Y, Ding G*, et al.* Dexamethasone attenuated bupivacaine-induced neuron injury in vitro through a threonine-serine protein kinase B-dependent mechanism. *Neuroscience* 2010, **167**(2)**:** 329-342.
