# Supplementary figures and images for: HSPA12A attenuates lipopolysaccharide-induced liver injury through inhibiting caspase-11-mediated hepatocyte pyroptosis via PGC-1α-dependent acyloxyacyl hydrolase expression
Source: Cell Death Differ. 2020 Apr 24;27(9):2651–67. doi: 10.1038/s41418-020-0536-x (PMC7429872; doi:10.1038/s41418-020-0536-x)

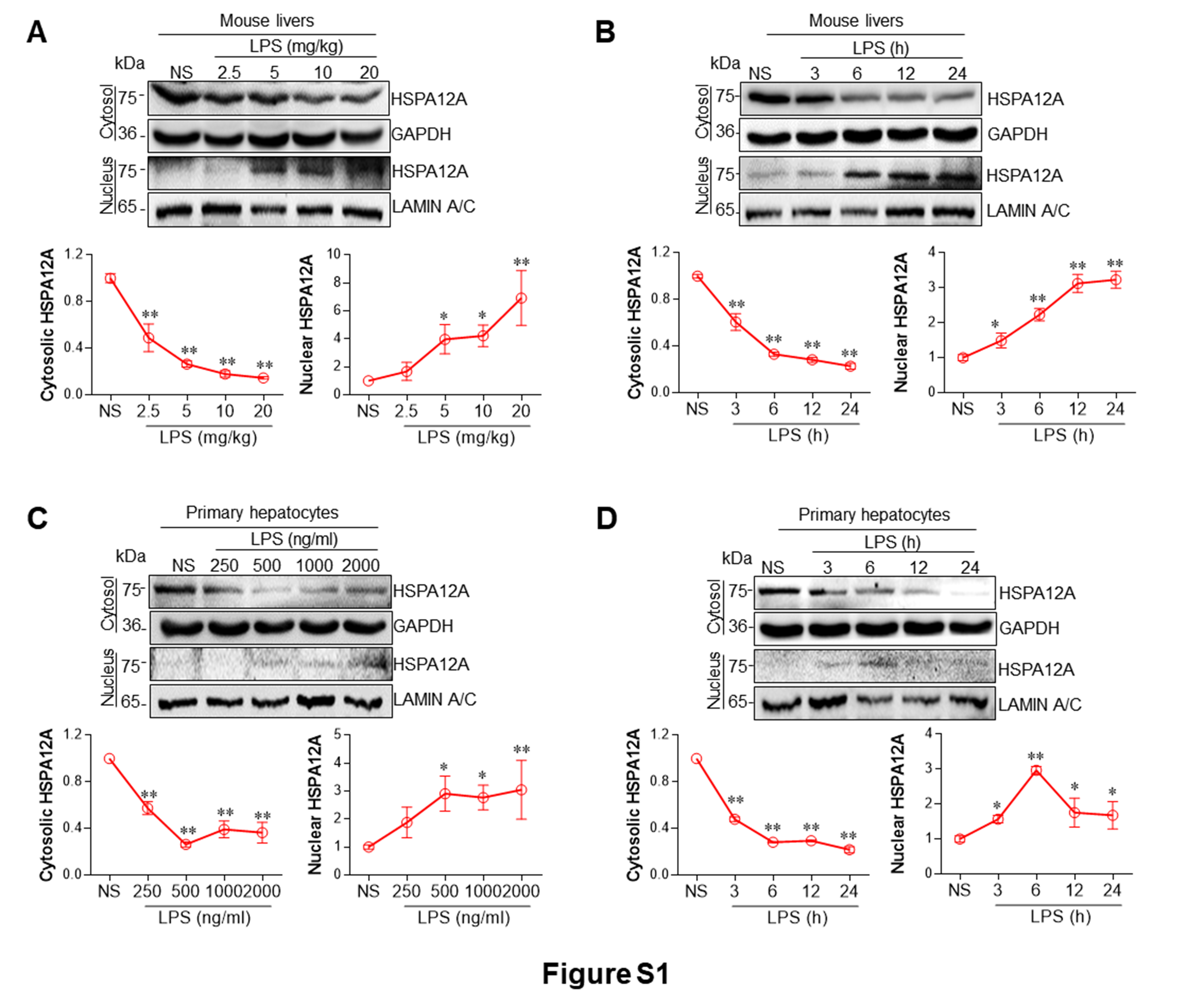

Supplement: Supplementary file 2 — Supplementary Figure S1 [file 41418_2020_536_MOESM2_ESM.tif]

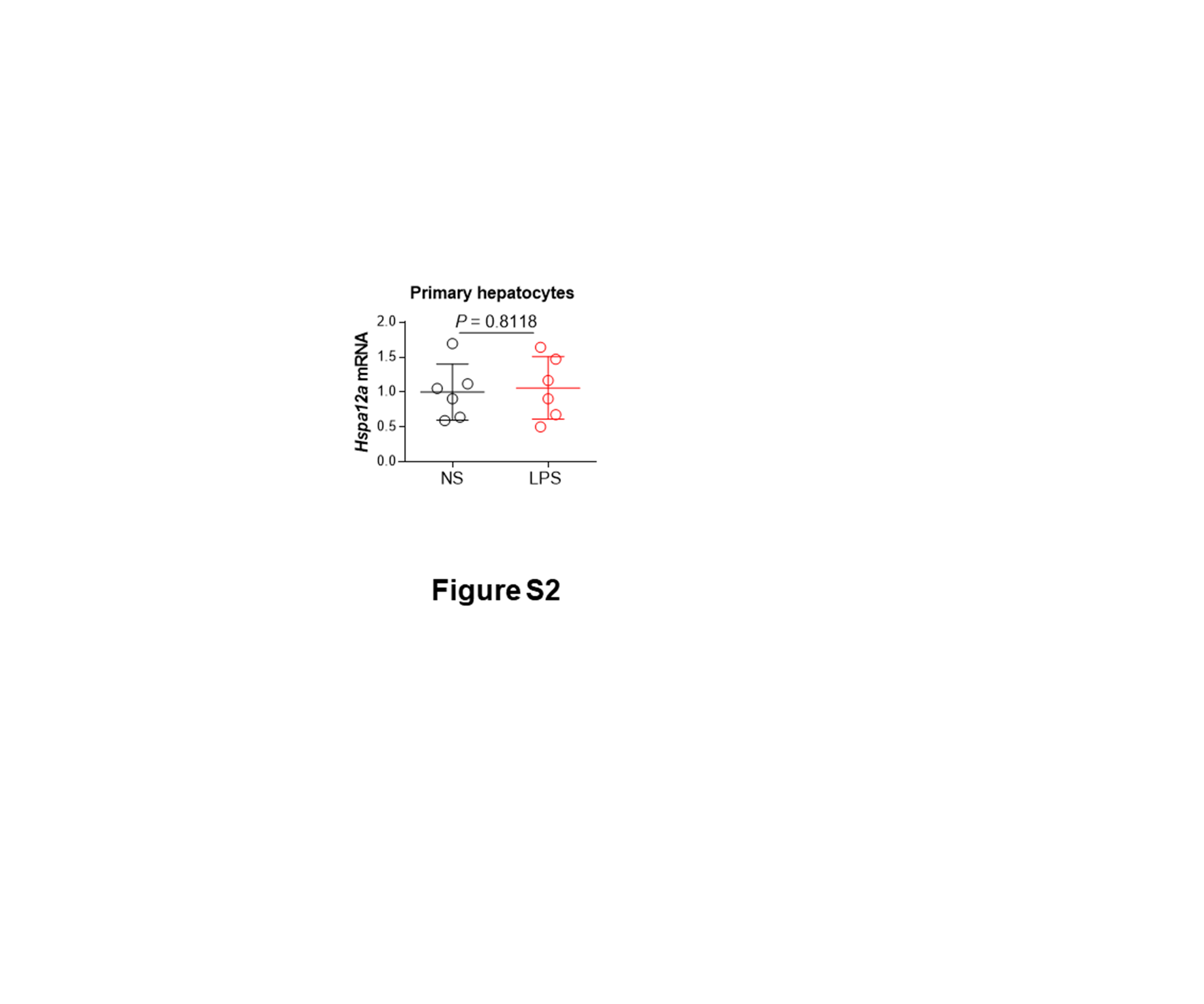

Supplement: Supplementary file 3 — Supplementary Figure S2 [file 41418_2020_536_MOESM3_ESM.tif]

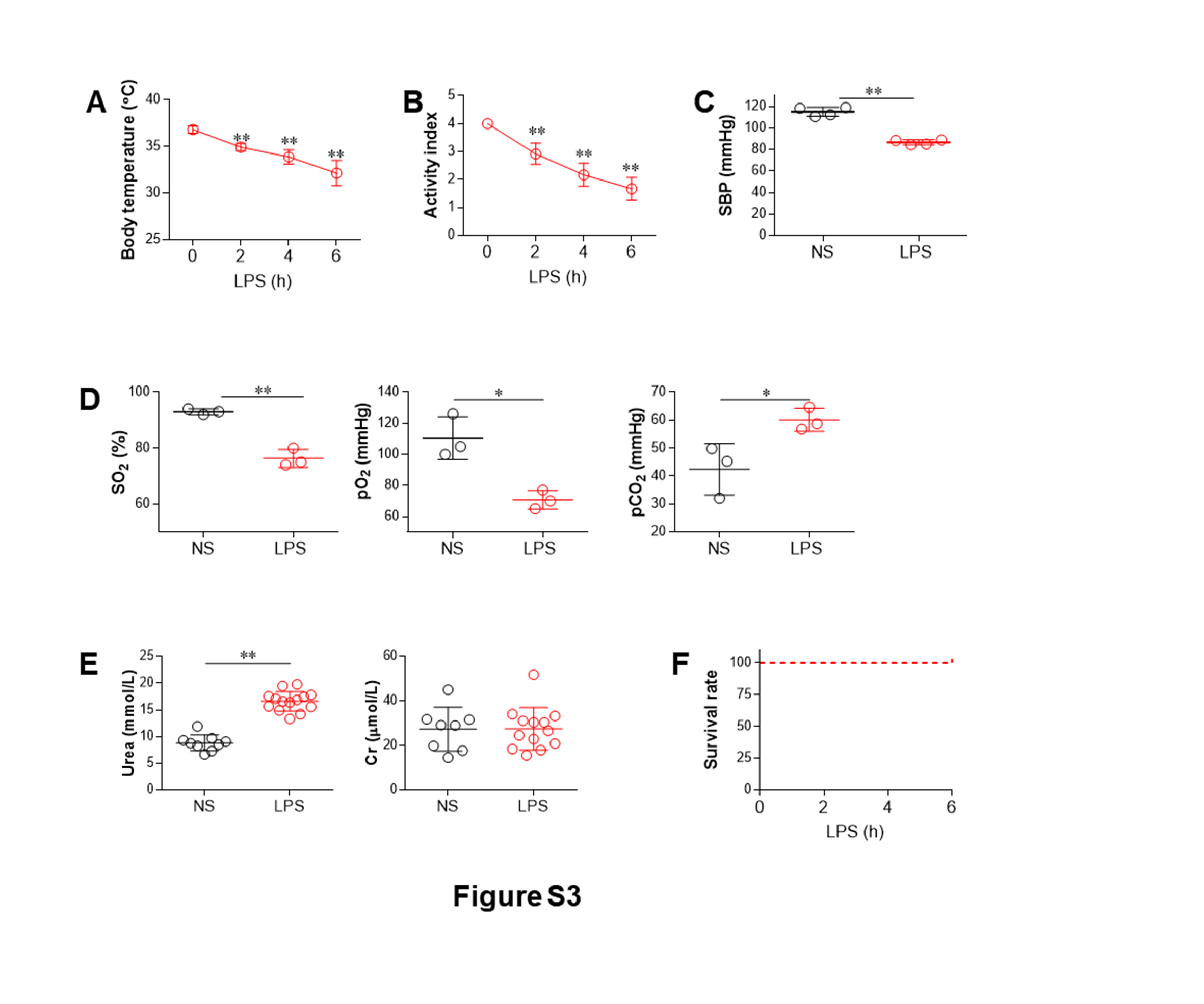

Supplement: Supplementary file 4 — Supplementary Figure S3 [file 41418_2020_536_MOESM4_ESM.tif]

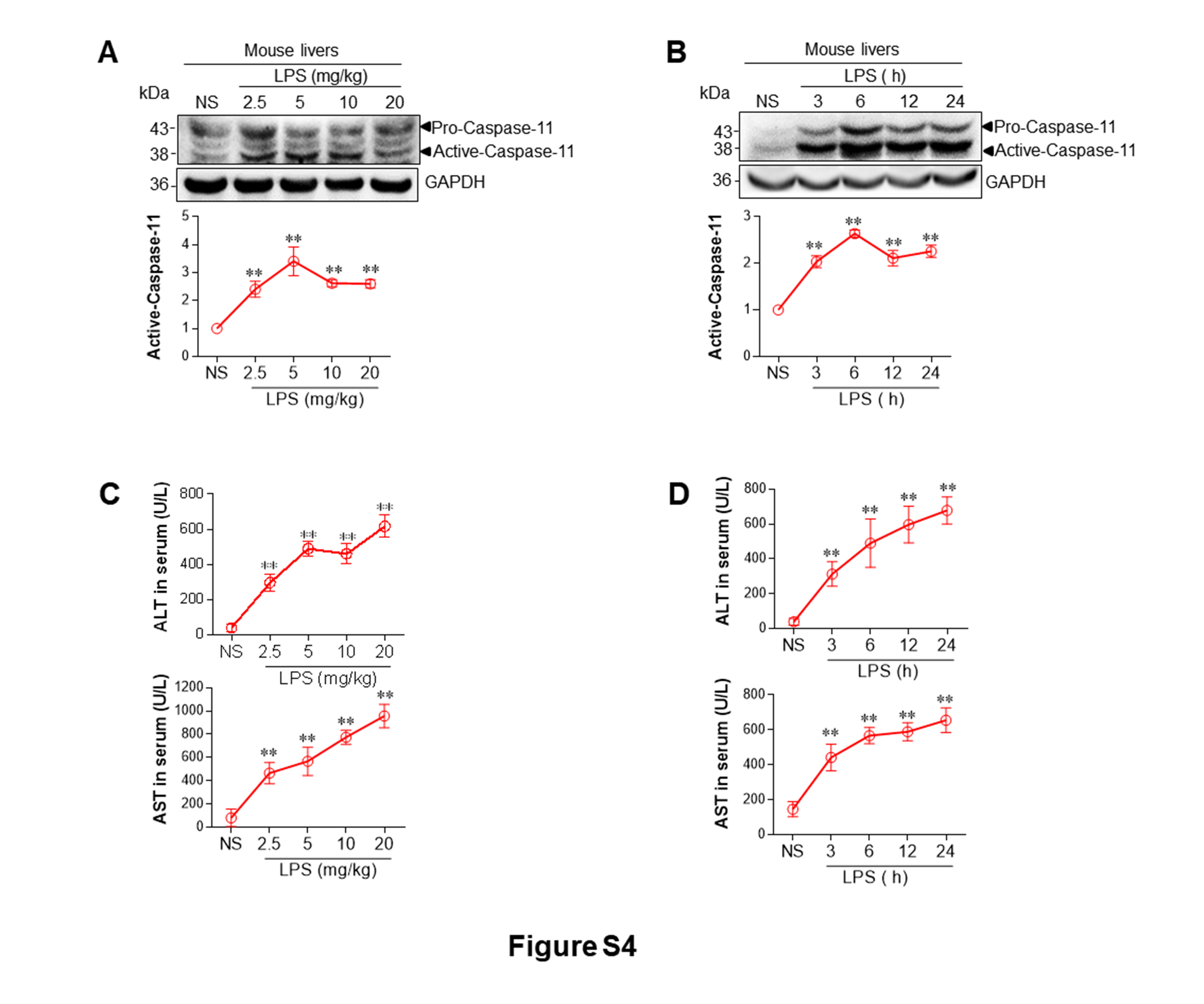

Supplement: Supplementary file 5 — Supplementary Figure S4 [file 41418_2020_536_MOESM5_ESM.tif]

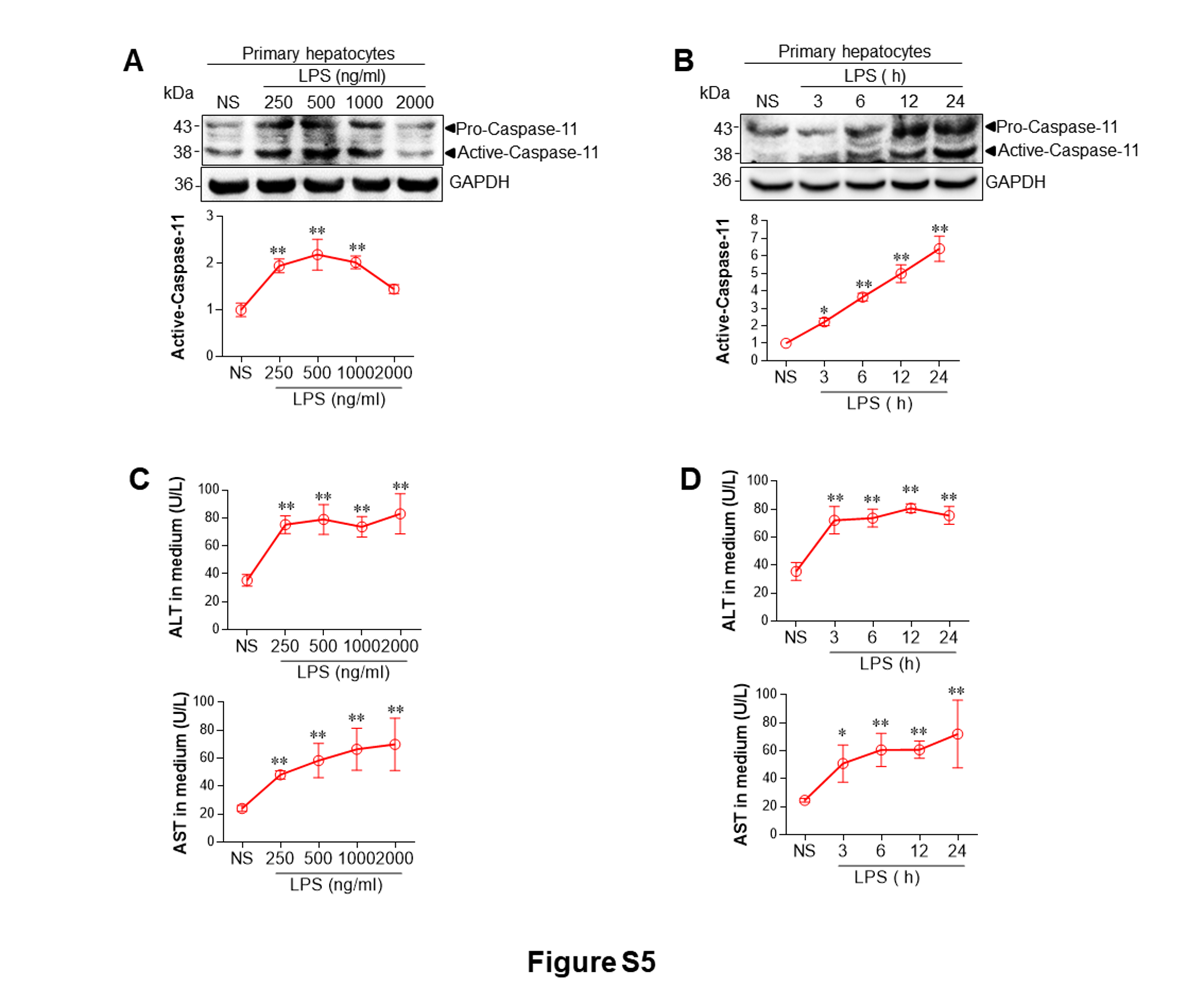

Supplement: Supplementary file 6 — Supplementary Figure S5 [file 41418_2020_536_MOESM6_ESM.tif]

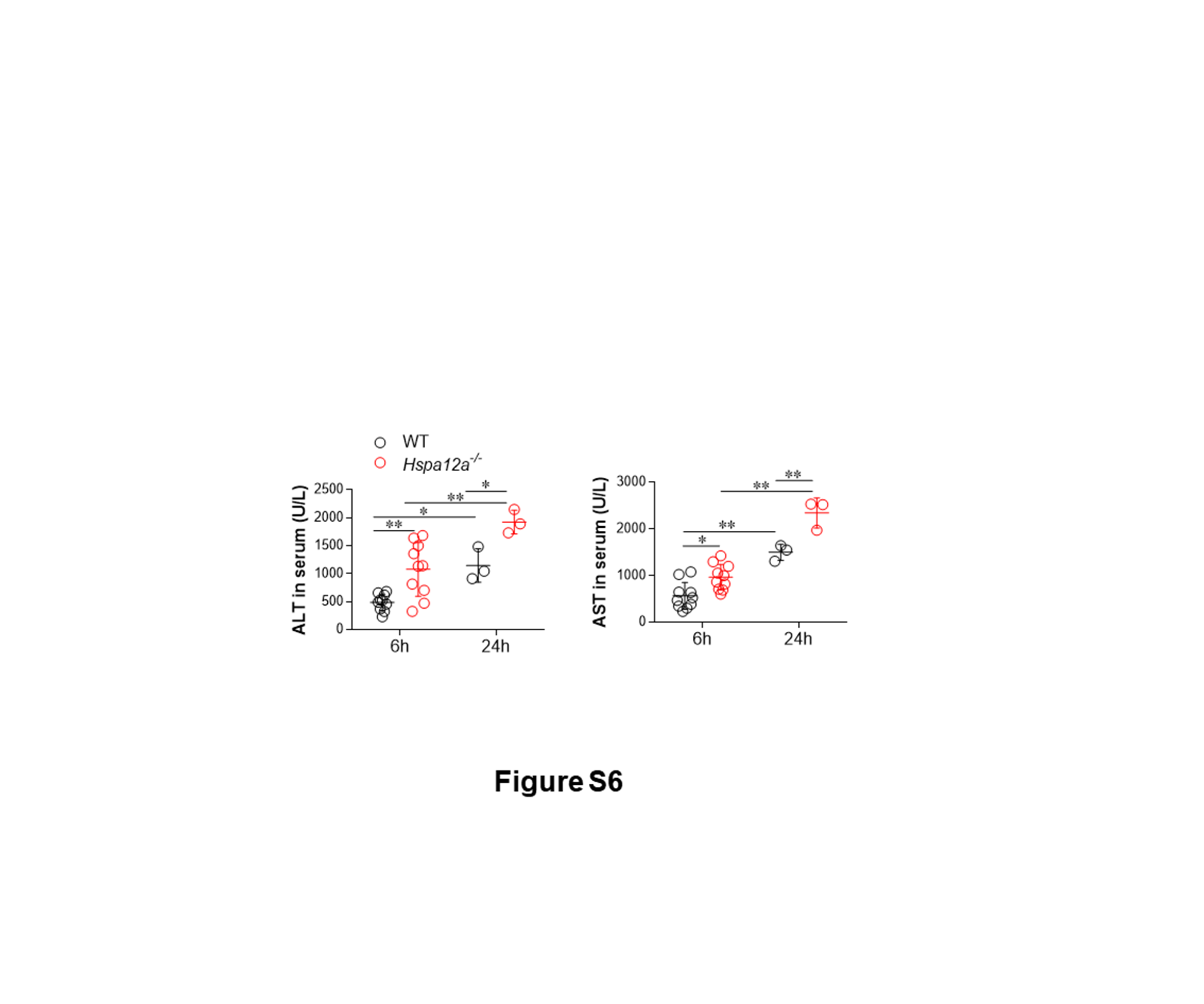

Supplement: Supplementary file 7 — Supplementary Figure S6 [file 41418_2020_536_MOESM7_ESM.tif]

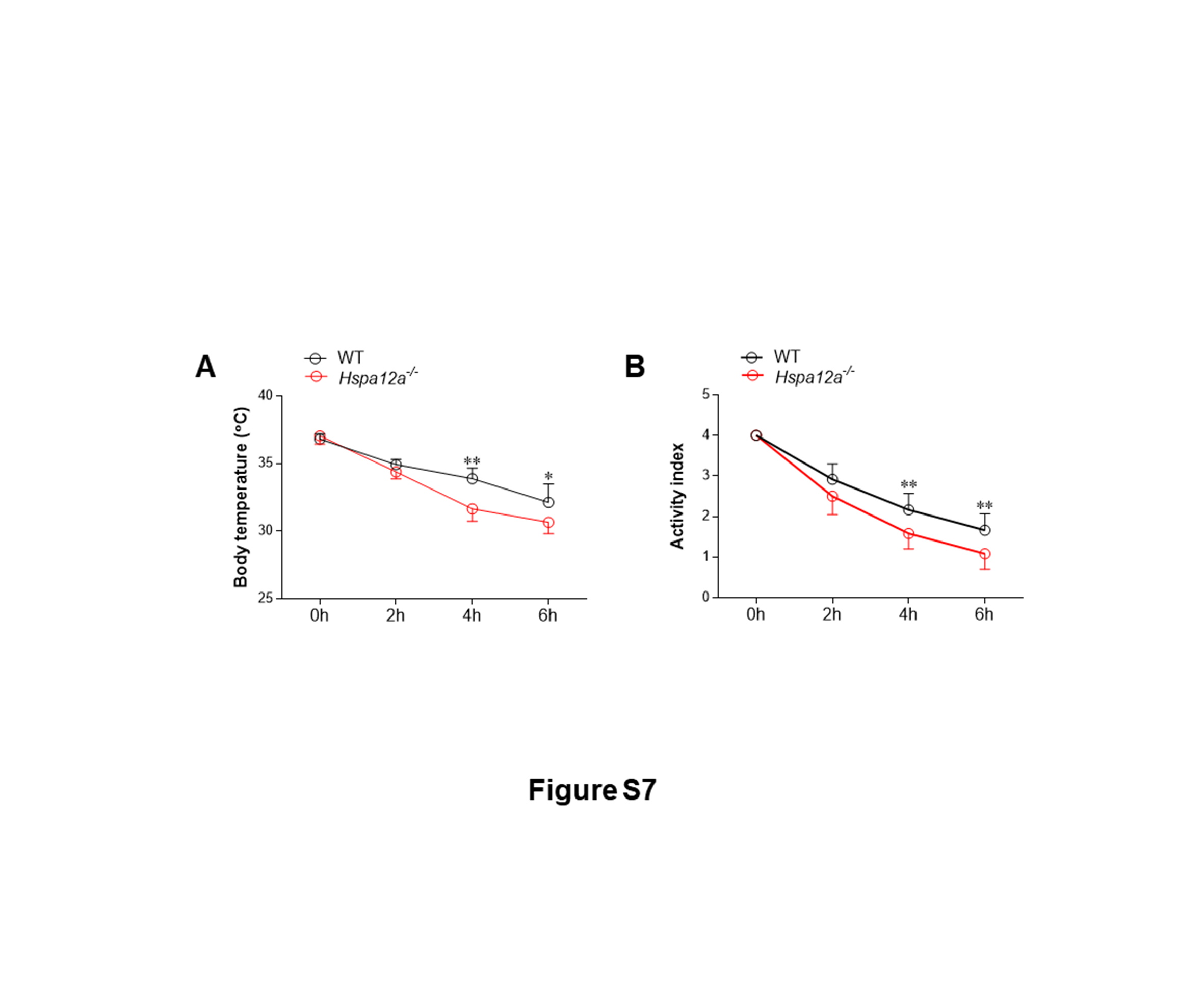

Supplement: Supplementary file 8 — Supplementary Figure S7 [file 41418_2020_536_MOESM8_ESM.tif]

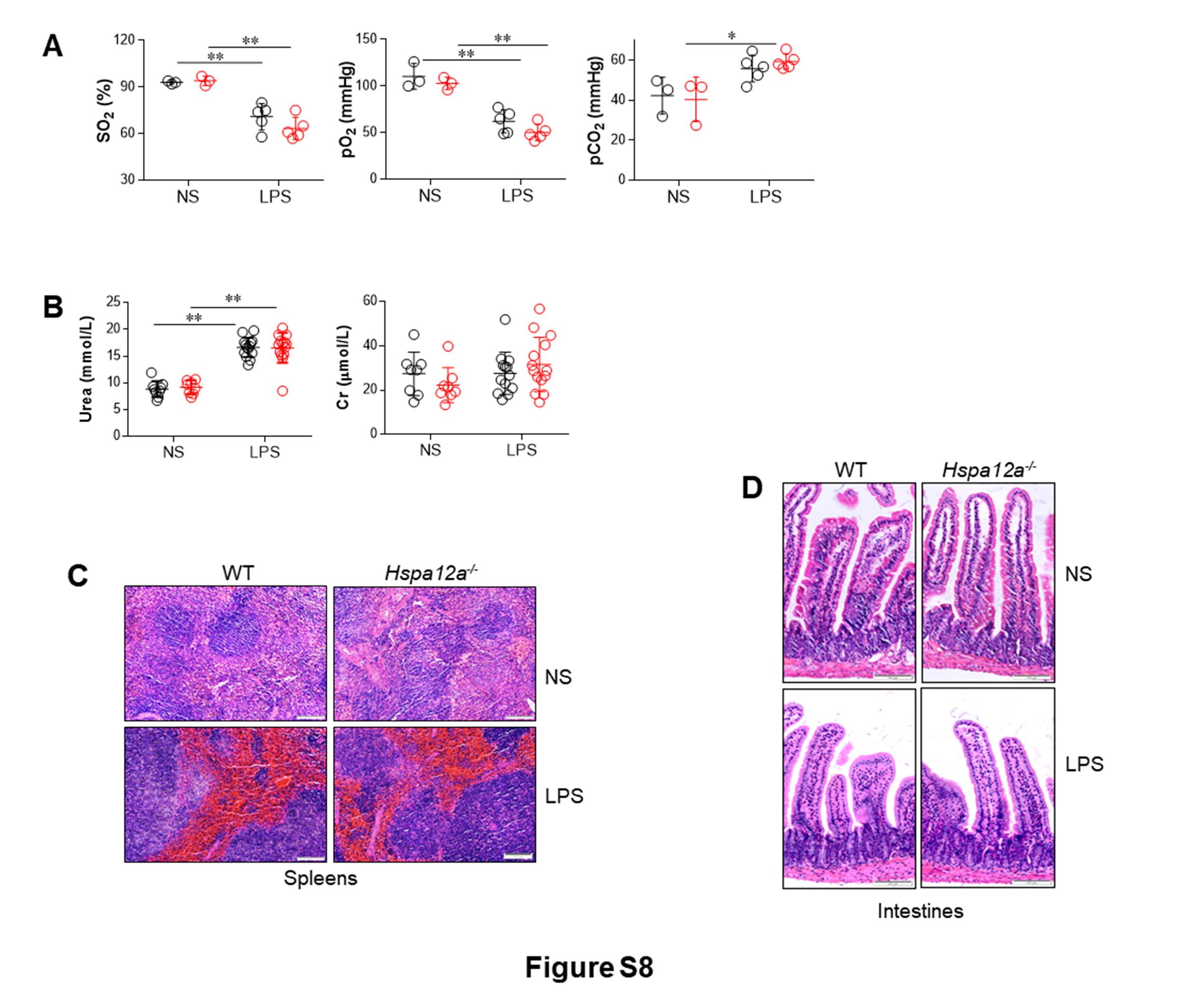

Supplement: Supplementary file 9 — Supplementary Figure S8 [file 41418_2020_536_MOESM9_ESM.tif]

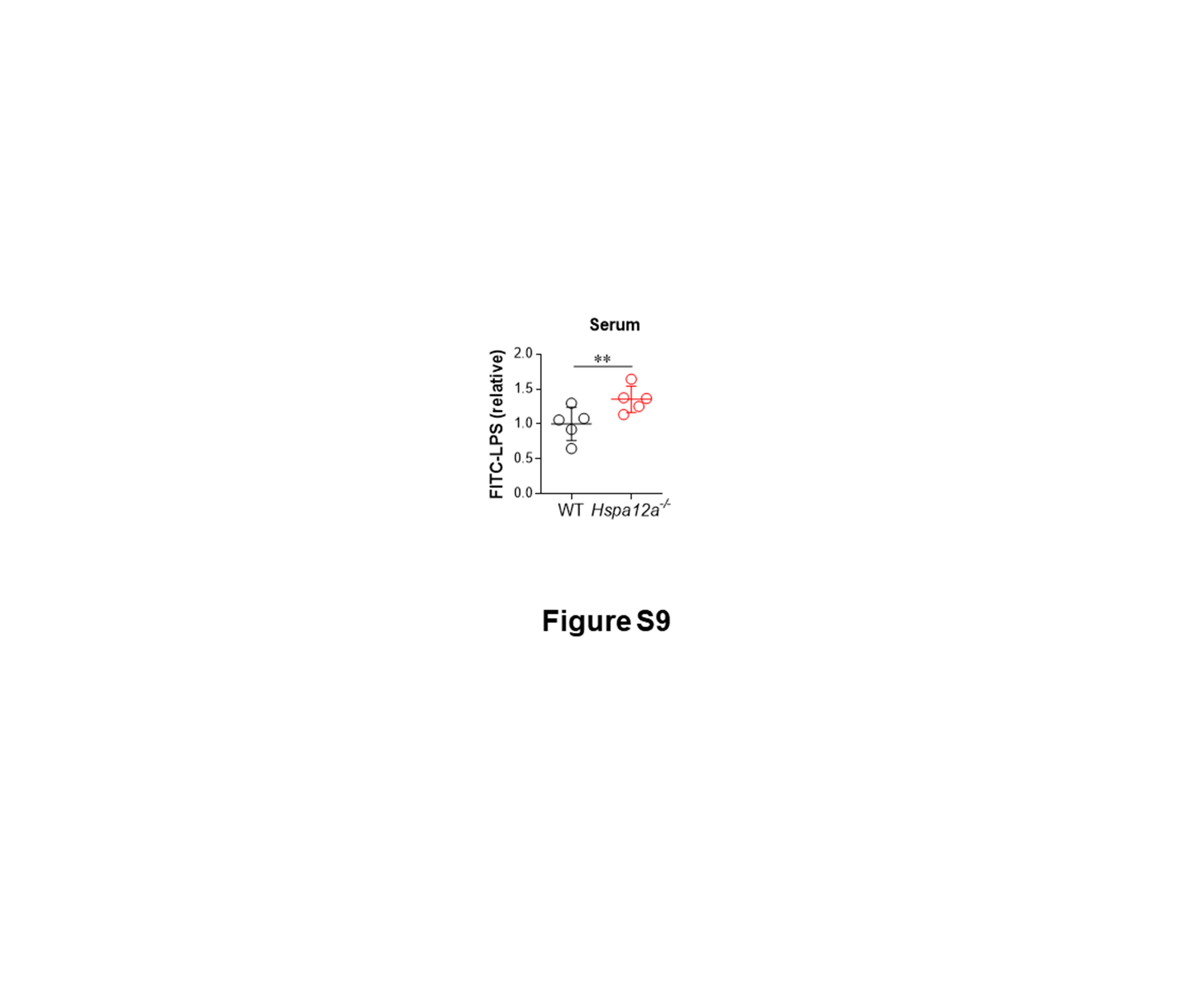

Supplement: Supplementary file 10 — Supplementary Figure S9 [file 41418_2020_536_MOESM10_ESM.tif]

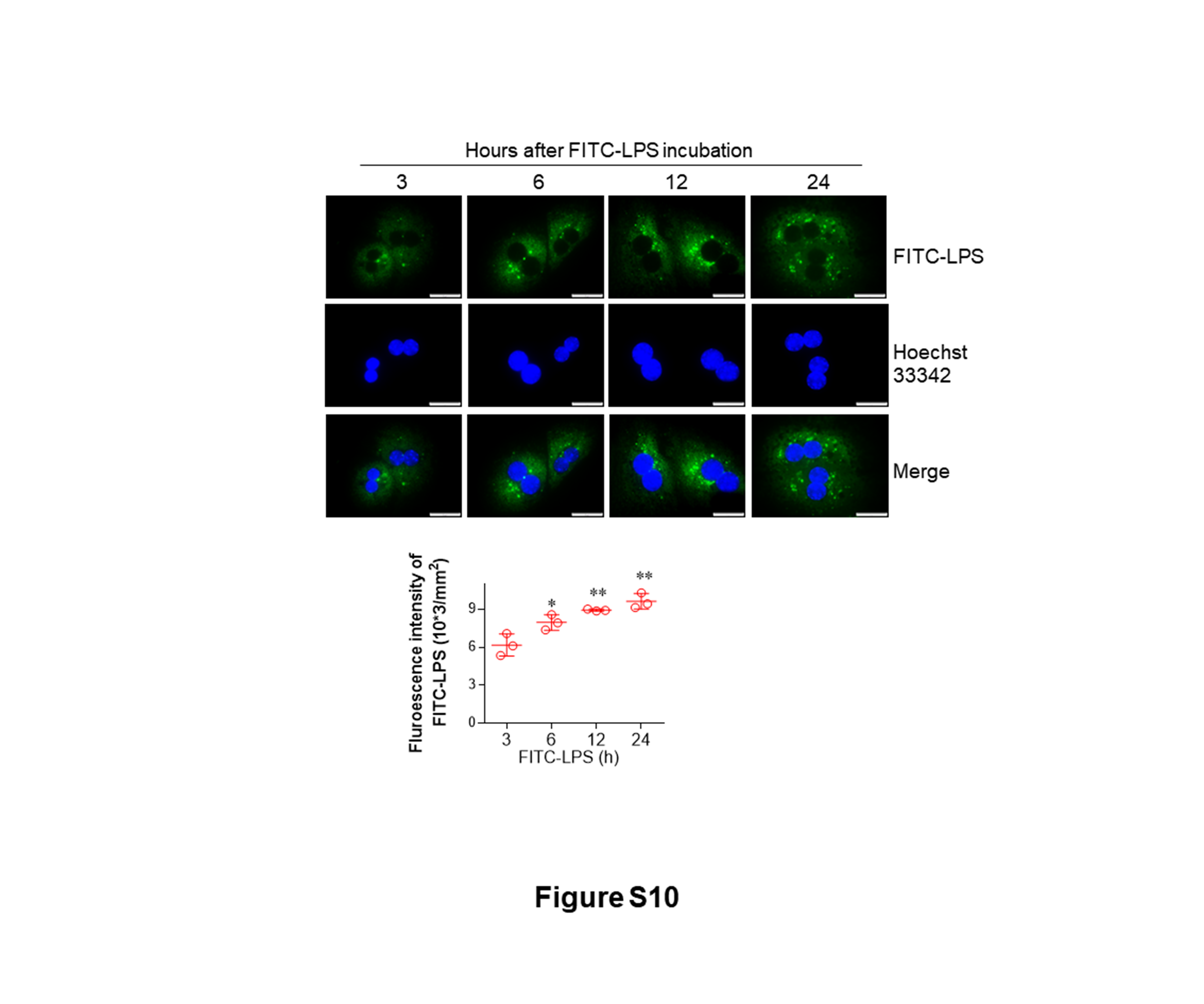

Supplement: Supplementary file 11 — Supplementary Figure S10 [file 41418_2020_536_MOESM11_ESM.tif]

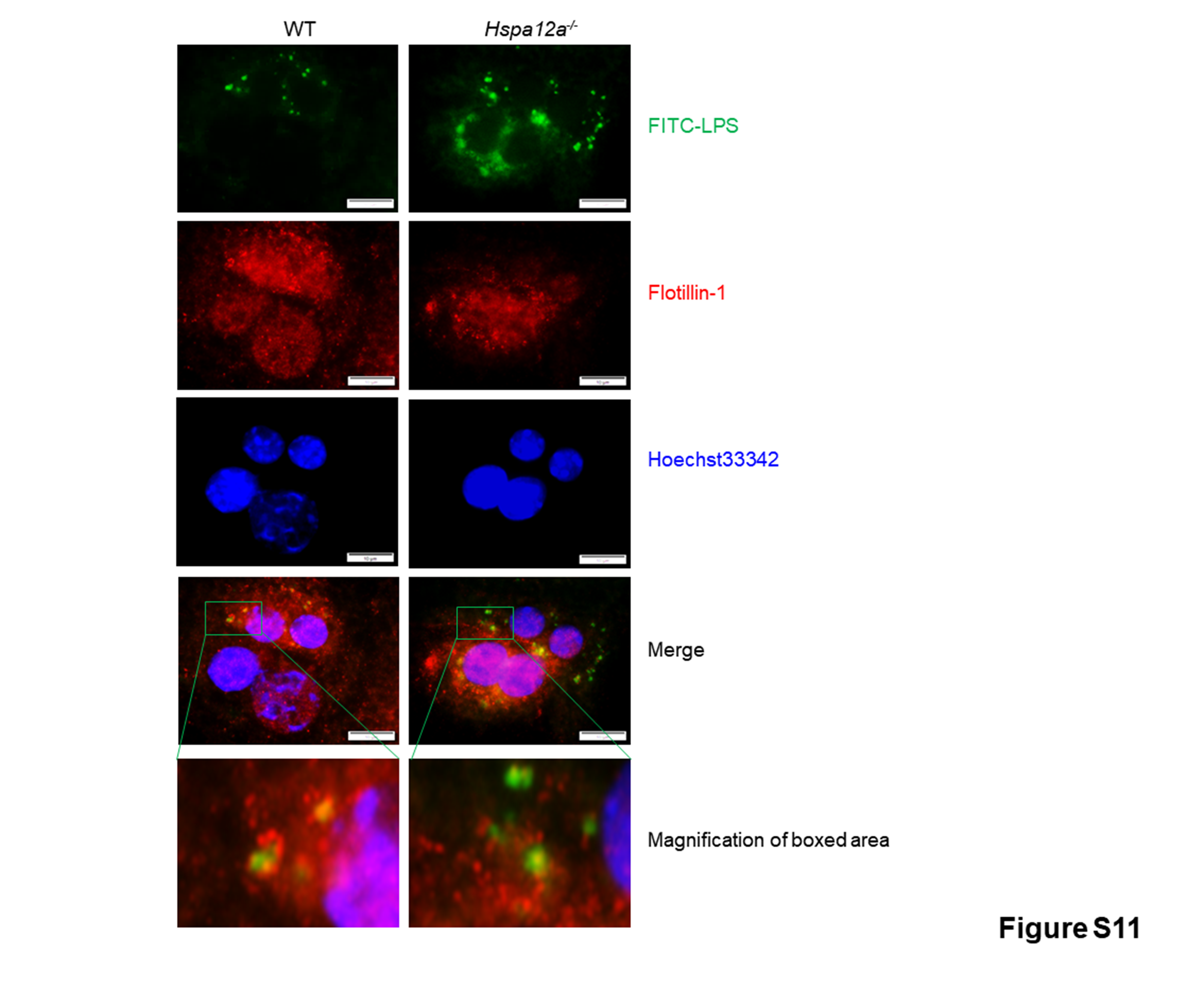

Supplement: Supplementary file 12 — Supplementary Figure S11 [file 41418_2020_536_MOESM12_ESM.tif]

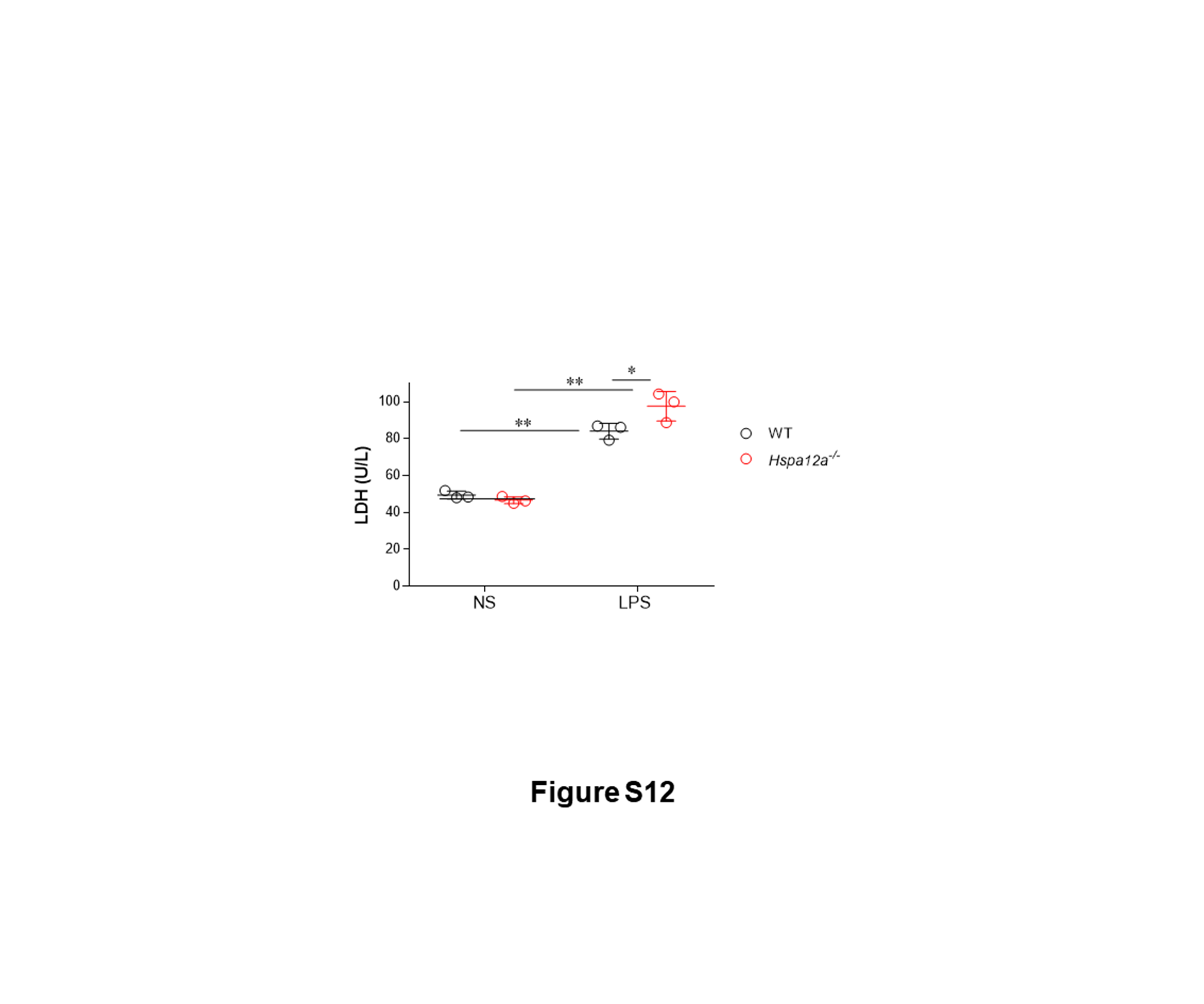

Supplement: Supplementary file 13 — Supplementary Figure S12 [file 41418_2020_536_MOESM13_ESM.tif]

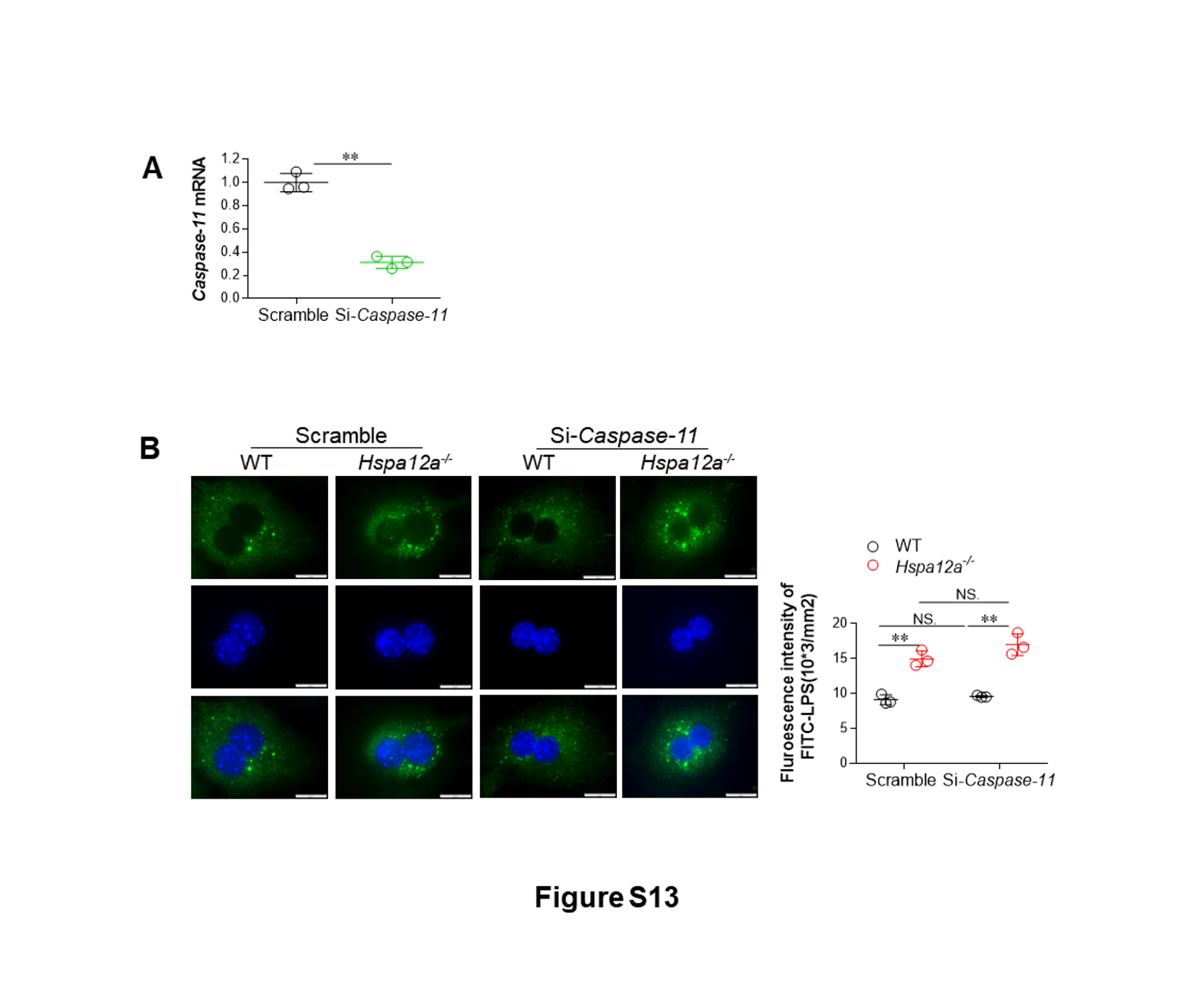

Supplement: Supplementary file 14 — Supplementary Figure S13 [file 41418_2020_536_MOESM14_ESM.tif]

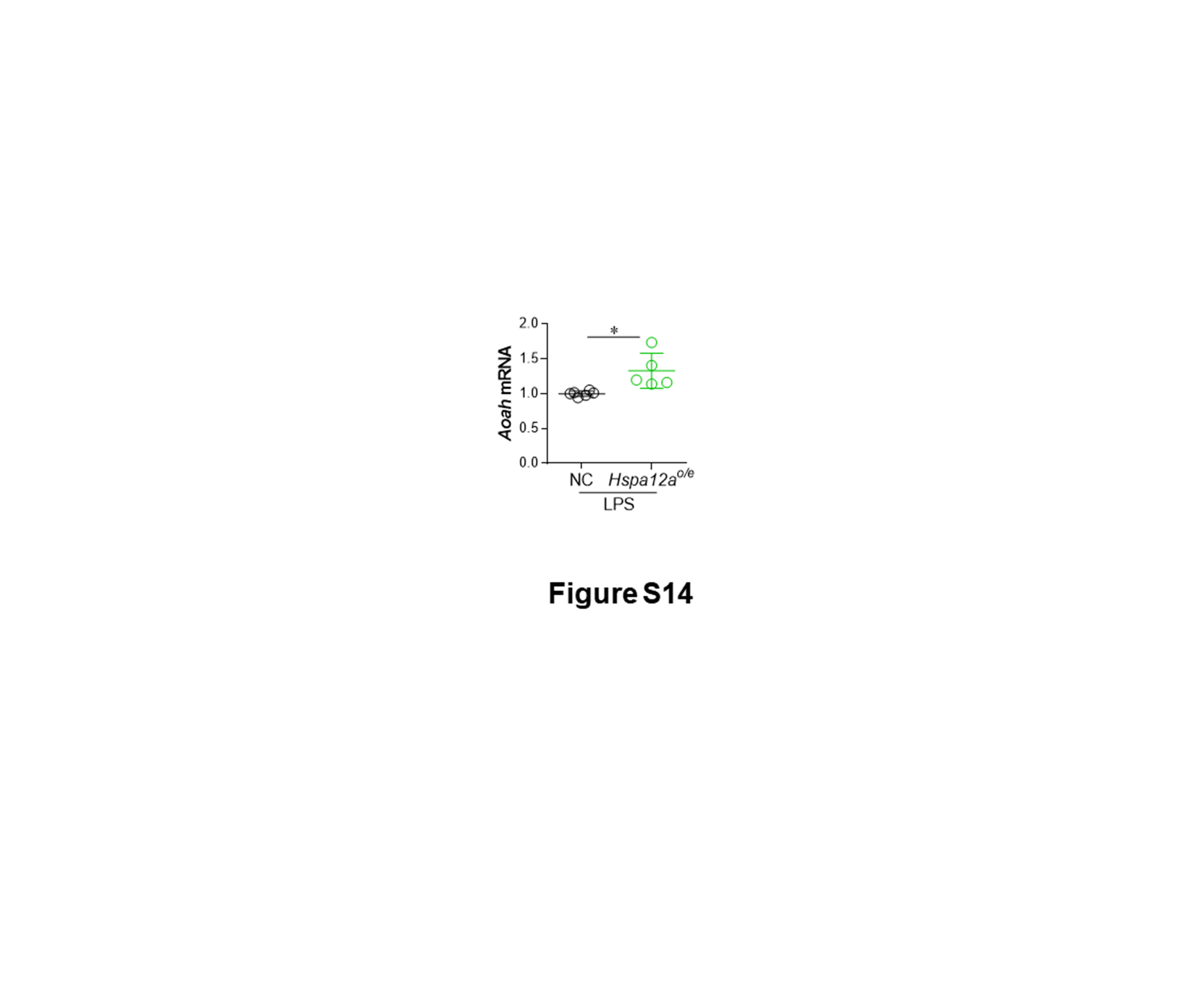

Supplement: Supplementary file 15 — Supplementary Figure S14 [file 41418_2020_536_MOESM15_ESM.tif]

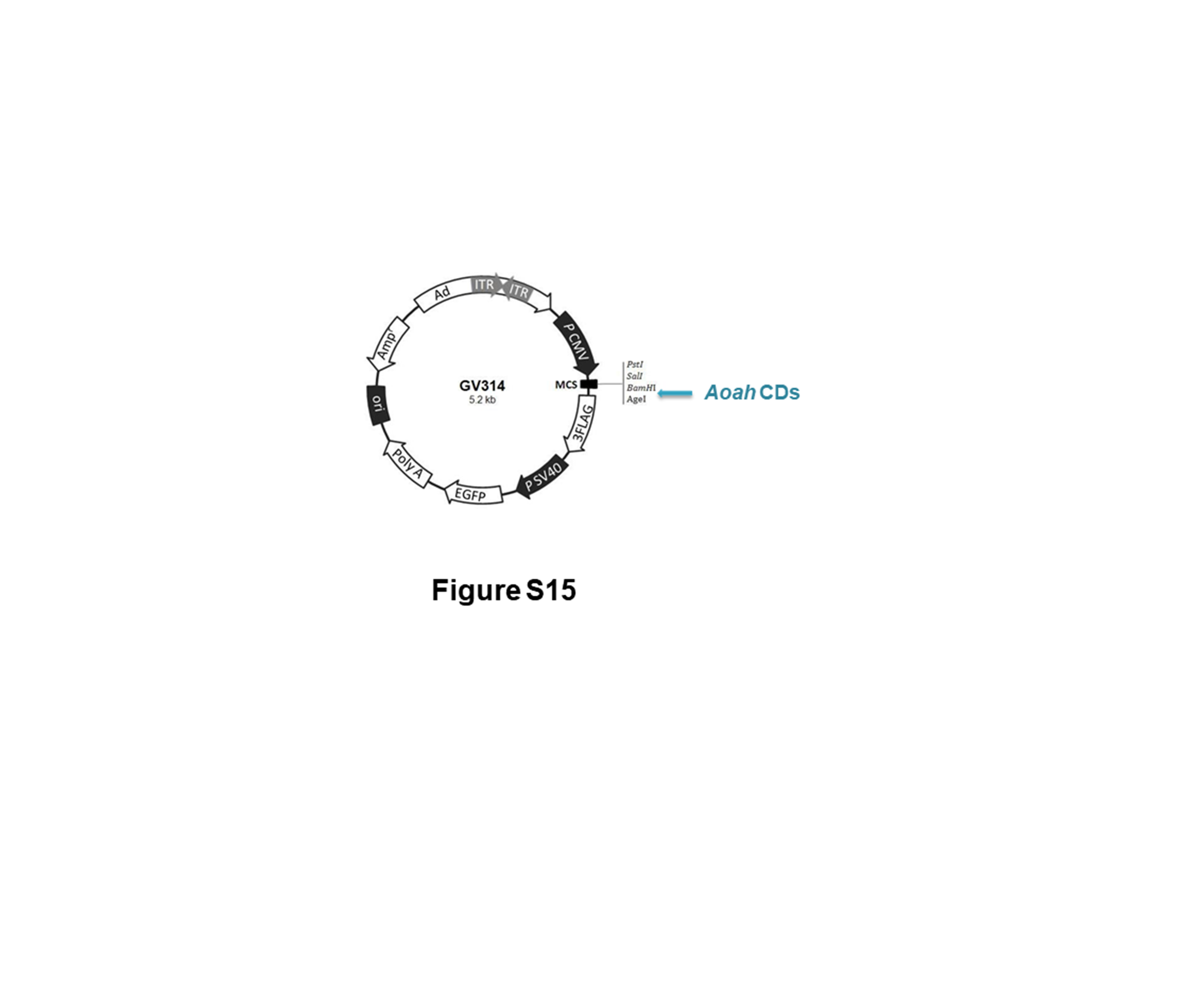

Supplement: Supplementary file 16 — Supplementary Figure S15 [file 41418_2020_536_MOESM16_ESM.tif]
