# Supplementary material for: HSPA12A attenuates lipopolysaccharide-induced liver injury through inhibiting caspase-11-mediated hepatocyte pyroptosis via PGC-1α-dependent acyloxyacyl hydrolase expression
Source: Cell Death Differ. 2020 Apr 24;27(9):2651–67. doi: 10.1038/s41418-020-0536-x (PMC7429872; doi:10.1038/s41418-020-0536-x)
Supplement: Supplementary file 17 — supplemental tables [file 41418_2020_536_MOESM17_ESM.pdf]

Table S1. Antibodies used in the experiments

Antibody	Source	Company	Catalog No.
anti-HSPA12A	Rabbit	Abcam	ab200838
anti-Lamin A/C	Rabbit	Proteintech Group	10298-1-AP
Anti-Caspase-11	Rat	Novus Biologicals	NB-120-10454
Anti-GSDMD	Rabbit	Abcam	ab209845
Anti-AOAH	Rabbit	Abcam	ab222913
Anti-PGC-1α	Rabbit	Millipore	ST1204
Anti-C/EBPα	Rabbit	Cell Signaling	#8178
anti-PPARγ	Rabbit	Cell Signaling	#2435
Anti-αfetoprotein (AFP)	Rabbit	Proteintech Group	14550-1-AP
anti-F4/80	Rat	Abcam	ab6640
anti-Neutrophil	Rat	Abcam	Ab2557
anti-Flag	Mouse	Sigma-Aldrich	F1804
anti-IgG	Mouse	Biotechnology	sc-2025
anti-GAPDH	Rabbit	Bioworld Technology	AP0063
anti-Flotillin-1	Rabbit	Cell Signaling	#18634

Table S2. Primers used in the experiments

Species	Gene name		Sequence (5'-3')
mouse	*Hspa12a*	Forward	CACAGGGGTGAGTTGGTCTC
		Reverse	TGAGGAGCCTTCCAGGCTAT
	Actin	Forward	TGTTACCAACTGGGACGACA
		Reverse	TCTCAGCTGTGGTGGTGAAG
	Caspase-11	Forward	ACGATGTGGTGGTGAAAGAGGAGC
		Reverse	TGTCTCGGTAGGACAAGTGATGTGG
	Caspase-1	Forward	GACCGAGTGGTTCCCTCAAG
		Reverse	GACGTGTACGAGTGGGTGTT
	Nlrp1	Forward	GGACCTCATGGTGGTTACTTTC
		Reverse	TCCCAGGGGCCGTAAACTT
	Nlrp3	Forward	ATTACCCGCCCGAGAAAGG
		Reverse	TCGCAGCAAAGATCCACACAG
	Asc	Forward	GGACAGTACCAGGCAGTTCG
		Reverse	GTCACCAAGTAGGGCTGTGT
	Tnfα	Forward	TCTCATTCCTGCTTGTGGC
		Reverse	CACTTGGTGGTTTGCTACG
	Il8	Forward	CAAGGCTGGTCCATGCTCC
		Reverse	TGCTATCACTTCCTTTCTGTTGC

Table S3. SiRNA sequence used in the experiments

Species	Gene name		Sequence (5'-3')
mouse	*Aoah*	Sense	CCACCGAGACGUUUGCAAATT
		Antisense	UUUGCAAACGUCUCGGUGGTT
mouse	*Caspase11*	Sense	ACACAGCAAAGUAGGUGAATT
		Antisense	UUCACCUACUUUGCUGUGUTT
